# GPCR19 Regulates P2X7R-Mediated NLRP3 Inflammasomal Activation of Microglia by Amyloid β in a Mouse Model of Alzheimer’s Disease

**DOI:** 10.3389/fimmu.2022.766919

**Published:** 2022-04-06

**Authors:** Jahirul Islam, Jung-Ah Cho, Ju-yong Kim, Kyung-Sun Park, Young-Jae Koh, Chu Young Chung, Eun-Jae Lee, Soo Jeong Nam, Kyoungyul Lee, Seoung-Heon Kim, Sung-Hye Park, Dong Young Lee, Byeong C. Kim, Kyung-Hwa Lee, Seung-Yong Seong

**Affiliations:** ^1^ Wide River Institute of Immunology, Seoul National University College of Medicine, Seoul, South Korea; ^2^ Department of Biomedical Sciences, Seoul National University College of Medicine, Seoul, South Korea; ^3^ Department of Inflammation, Shaperon Inc. Ltd, Seoul, South Korea; ^4^ Department of Neurology, Asan Medical Center, College of Medicine, University of Ulsan, Seoul, South Korea; ^5^ Department of Pathology, Asan Medical Center, Seoul, South Korea; ^6^ Department of Pathology, Kangwon National University Hospital, Kangwon National University School of Medicine, Chuncheon, South Korea; ^7^ Department of Neurology, Kangwon National University Hospital, Kangwon National University School of Medicine, Chuncheon, South Korea; ^8^ Department of Pathology, Seoul National University College of Medicine, Seoul, South Korea; ^9^ Department of Neuropsychiatry, Seoul National University College of Medicine, Seoul, South Korea; ^10^ Department of Neurology, Chonnam National University Medical School, Gwangju, South Korea; ^11^ Department of Pathology, Chonnam National University Hwasun Hospital and Medical School, Gwangju, South Korea; ^12^ Department of Microbiology and Immunology, Seoul National University College of Medicine, Seoul, South Korea

**Keywords:** taurodeoxycholate, GPCR19, P2X7R, Alzheimer’s disease, neuroinflammation, inflammasome

## Abstract

Amyloid β (Aβ) and/or ATP activate the NLRP3 inflammasome (N3I) *via* P2X7R in microglia, which is crucial in neuroinflammation in Alzheimer’s disease (AD). Due to polymorphisms, subtypes, and ubiquitous expression of P2X7R, inhibition of P2X7R has not been effective for AD. We first report that taurodeoxycholate (TDCA), a GPCR19 ligand, inhibited the priming phase of N3I activation, suppressed P2X7R expression and P2X7R-mediated Ca^++^ mobilization and N3I oligomerization, which is essential for production of IL-1β/IL-18 by microglia. Furthermore, TDCA enhanced phagocytosis of Aβ and decreased the number of Aβ plaques in the brains of 5x Familial Alzheimer’s disease (5xFAD) mice. TDCA also reduced microgliosis, prevented neuronal loss, and improved memory function in 5xFAD mice. The pleiotropic roles of GPCR19 in P2X7R-mediated N3I activation suggest that targeting GPCR19 might resolve neuroinflammation in AD patients.

## Introduction

Despite Alzheimer’s disease (AD) being the most common cause of dementia, no effective treatments are currently available (1). Cholinergic, tau, and amyloid hypotheses have been suggested to explain the pathophysiology of AD (2). Currently, the treatments of choice for AD patients are mostly based on cholinergic neurotransmission, which does not sufficiently mitigate the progression of AD (3). Although glial cells constitute more brain cells than neurons, neurons have long received more attention than have glial cells, possibly due to the prominent neurological symptoms of AD patients (4). Within the last few years, however, clinical trials have moved to reduce neuroinflammation induced by reactive microglia (5).

Neuroinflammation led by Aβ-activated microglia induces neuronal apoptosis in the hippocampus and cortex of AD patients (6). In the brains of AD patients, proinflammatory mediators, such as reactive oxygen species (ROS), reactive nitrogen species (RNS), IL-1β, IL-6, and tumor necrosis factor (TNF)-α, are frequently increased (7). The insoluble aggregates of Aβ and hyperphosphorylated tau, which make neurofibrillary tangles, are crucial for neuroinflammation in these patients (8). Damage-associated molecular patterns (DAMPs), such as Aβ aggregates and hyperphosphorylated tau, interact with pattern recognition receptors (PRRs) on brain cell membranes or in the cytosol to initiate proinflammatory pathways (9). Sustained neuronal apoptosis may unleash more DAMPs in the brain, which further amplifies sterile inflammation in the brain (10). Considering that neuroinflammation plays crucial roles in cognitive and memory deficits through neuronal loss, controlling neuroinflammation may provide promising therapeutic strategies (11).

Inflammasome plays a central role in the pathogenesis of many inflammatory disorders, including AD (12). The NACHT, LRR, and PYD domains containing protein 3 (NLRP3) or NLRP1 polymorphisms are closely related to AD incidence (13, 14). Among several inflammasomes, the most crucial contributor in AD pathologies is NLRP3 (15). Aβ was efficiently cleared and cognition was improved in AD mice by inhibiting activation of the NLRP3 inflammasome (N3I) (16), suggesting that N3I is crucial in inflammatory neurodegeneration in AD (17).

The PRRs, such as CD36, CD14, TLR2, RAGE, and P2X7R, of microglia are crucial in Aβ-triggered activation of the NLRP1 inflammasome (N1I) (14) and N3I (18), which causes IL-1β/IL-18/pyroptosis-mediated inflammation in mice and in AD patients (13).

Unfortunately, however, strategies targeting components of N3I have not been successful in clinical trials for AD until now (19). This may be due to the redundant proinflammatory pathways activated by Aβ. For example, several PRRs recognize cytoplasmic tau and extracellular Aβ. Furthermore, the inflammasome is activated by canonical and noncanonical pathways, consisting of pro‐caspase‐1/4/5/11, gasdermin D, ASC, NLR proteins (such as NLRP1, NLRP3, NLRC4, NLRP6, or NLRP12), absent in melanoma 2 (AIM2), IFN‐inducible protein 16 (IF116), and pyrin (20, 21), although their roles in Aβ-mediated inflammasomal activation have not yet been elucidated in detail. The significance of redundancy in developing inflammasomal inhibitors was well demonstrated in studies using the NLRP3-specific inhibitor MCC950 (22). MCC950 showed promising efficacy in preclinical settings but not in clinical settings (16). The redundancy of inflammasomal activation pathways implies the evolutionary significance of inflammasomes responding to diverse environmental or endogenous threats to maintain tissue homeostasis (23).

For these reasons, molecules at higher levels of the inflammasomal signaling cascade need to be regulated to overcome the redundancy in inflammasomal activation. A more plausible approach may involve targeting P2X7R, which is one of the top regulators of the signaling cascade necessary for N3I activation (24). Brain cells, including microglia, express purinergic receptors, and both ionotropic P2X and metabotropic P2Y receptors are crucial in AD pathogenesis (25). P2X7R is the ion channel primarily studied in terms of N3I activation by Aβ (26). Upon binding with ATP or Aβ, P2X7R renders cell membranes permeable to K^+^ and Ca^++^, which activate the inflammasome (27). N3I of P2X7R^-/-^ microglia was not activated in response to Aβ (28), suggesting the essential role of P2X7R in Aβ-mediated neuroinflammation. P2X7R promotes the assembly of N3I, secretion of IL1β/18, and pyroptosis (29). Intriguingly, P2X7R is overexpressed in glial cells from AD patients, and Aβ injection into the hippocampus increases P2X7R expression (30). Taken together, P2X7R plays a key role in chronic neuroinflammation and neurodegeneration in AD.

Apyrase blocks the activation of N3I by Aβ (31), suggesting that the ATP-P2X7R interaction is crucial in N3I activation in response to Aβ (32). High levels of ATP are passively released from necrotic cells and act as proinflammatory DAMPs, binding to P2X7R and activating N3I (33). P2X7R activation creates membrane pores through which ATP can leak further (34).

Activation of P2X7R is also crucial in impairing phagocytosis of Aβ (35). In AD patients, the phagocytic ability of microglia was insufficient to clear Aβ (5). P2X7R^-/-^ microglia phagocytosed Aβ more efficiently than wild-type microglia (36). Thus, many P2X7R inhibitors have been developed to control inflammasomal activation without success until now (37). These inhibitors effectively decreased inflammatory responses in AD mice (38). However, many of these did not meet the clinical needs (39). Human P2X7R is highly polymorphic and exists as several isotypes (40). Ten human P2X7R gene splice variants (P2X7RA–J) might produce a complex combination of P2X7R with various haplotypes that cause a broad spectrum of responsiveness to P2X7R inhibitors in clinical settings.

Bile acids (BAs) are primarily synthesized from cholesterol in the liver; however, intestinal bacteria produce secondary BAs from primary BAs (41). In addition to metabolic homeostasis, various bile acids play pivotal roles in modulating inflammation (42). For example, different GPCR19 agonists inhibit inflammation of the stomach (43) and brain (44). Activation of GPCR19 attenuates caspase-8/NLRP3-mediated neuroinflammation in rats (45). For these reasons, GPCR19, which is a nonpolymorphic physiological regulator of P2X7R, might be an alternative target to control N3I. We found that GPCR19 regulates P2X7R in N3I activation. In this study, we used taurodeoxycholic acid (TDCA), a GPCR19 agonist, because taurine-conjugated BAs activate GPCR19 (46) with less cytotoxicity than unconjugated BAs and glycine-conjugated BAs (47). TDCA inhibited the priming phase and activation phase in N3I activation of microglia by activating adenylate cyclase and inhibiting P2X7R, respectively.

## Results

### GPCR19 Is Colocalized With P2X7R in Microglia, and TDCA Regulates Their Expression

**Figure 1:**
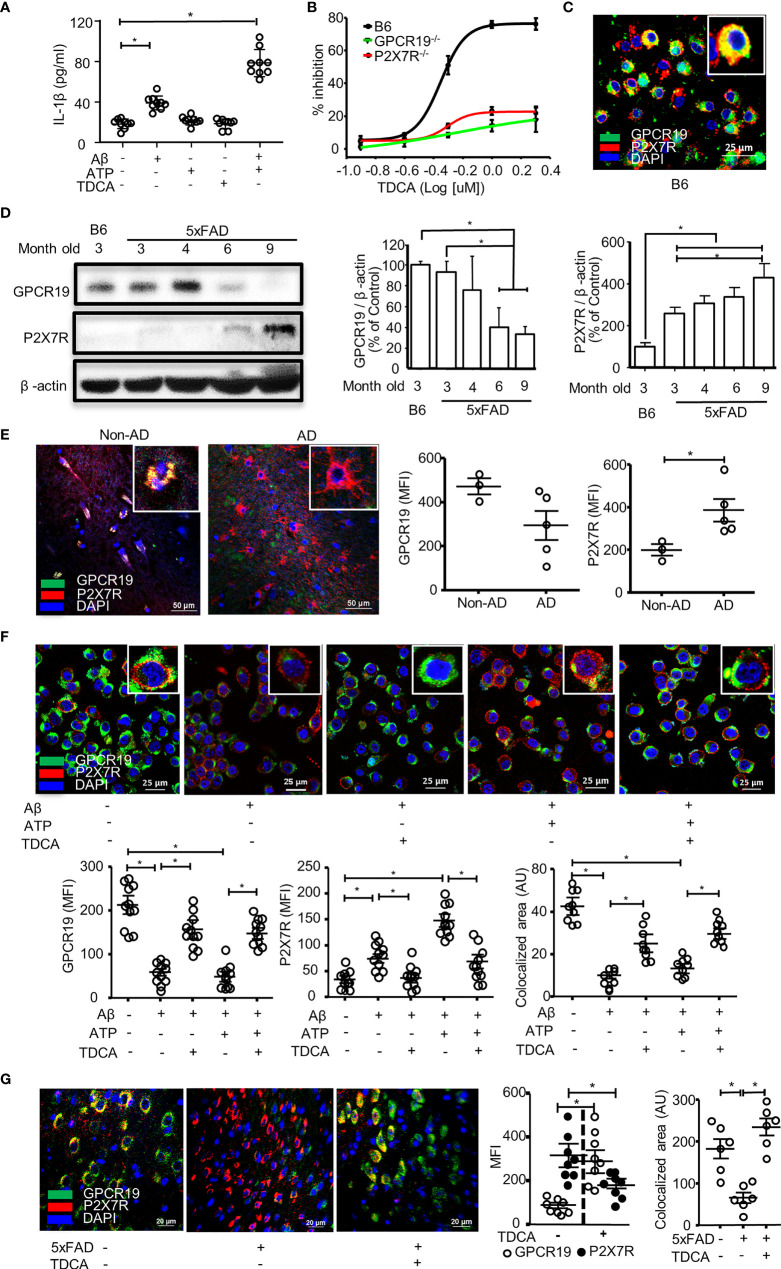
GPCR19 and P2X7R are necessary for the inhibition of IL-1β production in microglia by TDCA. **(A)**, Aβ ± ATP increased IL-1β production of BV2 cells when measured by ELISA. **(B)**, Primary microglia were isolated from wild-type B6, P2X7R^-/-^, and GPCR19^-/-^ mice and were treated with Aβ, ATP, and TDCA. IL-1β production by microglia was determined by ELISA. % Inhibition = (IL-1β concentration without TDCA - IL-1β concentration with TDCA)/(IL-1β concentration without TDCA) x 100. **(C)**, Colocalization of GPCR19 and P2X7R on membranes of primary microglia was tested using confocal microscopy. **(D)**, Protein expression levels of GPCR19 or P2X7R in the frontal cortex were analyzed using western blotting. The expression levels in B6 mice were considered 100% (n=3). **(E)**, Human brain sections from patients with AD or patients without AD (non-AD, n=3) were stained with anti-GPCR19 Ab (green) and anti-P2X7R Ab (Red). Average of five different ROIs (× 400) in each section were analyzed in the right panel. **(F)**, The expression levels of GPCR19 (green) and P2X7R (red) on the surface of BV2 cells were analyzed using confocal microscopy after treatment with Aβ, ATP, and TDCA for 1 (h) The areas of colocalization (yellow) of GPCR19 (green) and P2X7R (red) were calculated using ImageJ. **(G)**, Expression levels of GPCR19 and P2X7R in the frontal cortex of B6 (5xFAD -) or 5xFAD mice (5xFAD +) were analyzed using confocal microscopy after treatment with TDCA (1 mg/kg, i.p.) for 10 weeks. Colocalized areas of GPCR19 and P2X7R (yellow) were calculated using ImageJ. Aβ, ATP and TDCA were used at 2 µM, 1 mM and 400 ng/ml, respectively, throughout the study otherwise denoted. Data are from more than three independent experiments throughout the study otherwise denoted. The individual samples are shown with the mean ± SEM and *P < 0.05 using Student’s unpaired *t*-test throughout the study otherwise denoted.

First, we tested IL-1β secretion of BV2 microglial cells in response to Aβ (2 µM) ± ATP (1mM) or TDCA (400ng/ml) to optimize experimental settings (**Figure 1A**). IL-1β secretion was increased upon treatment with Aβ alone and was further increased when combined with ATP. However, ATP or TDCA alone did not increase IL-1β secretion. Primary microglia from the brains of C57BL/6 (B6), P2X7R^-/-^, or GPCR19^-/-^ mice were sorted using a magnetic column. The purity was above 95% (**Supplementary Figure 1A**). When stimulated with Aβ (2 µM) and ATP (1 mM), the IC_50_ of TDCA in inhibiting IL-1β secretion by microglia from B6 mice was 490.6 nM (**Figure 1B**). However, TDCA did not inhibit IL-1β secretion by microglia from GPCR19^-/-^ or P2X7R^-/-^ mice when stimulated with Aβ (2 µM) and ATP (1 mM) (**Figure 1B**), suggesting that GPCR19 might regulate P2X7R function. GPCR19 and P2X7R were colocalized in resting primary microglial membranes (**Figure 1C**; **Supplementary Figure 1B**). The expression levels of GPCR19 in the frontal cortex were significantly lower in 6- and 9-month-old 5xFAD mice than in 3-month-old 5xFAD mice or B6 mice (**Figure 1D**). In comparison, the expression levels of P2X7R in the frontal cortex were significantly higher in 9-month-old 5xFAD mice than that of 3-month-old 5xFAD mice or B6 mice (**Figure 1D**). Brain tissues from individuals with AD or without AD were analyzed after immunohistochemical staining. GPCR19 and P2X7R were colocalized in the brain cells of non-AD subjects (**Figure 1E**; **Supplementary Figure 2**). Expression of GPCR19 in non-AD subjects (n = 3) was higher than those of AD patients (n = 5). On the contrary, expression of P2X7R in non-AD subjects was lower than those of AD patients. Treatment of BV2 cells with Aβ ± ATP for 1 h significantly downregulated the expression of GPCR19 on the cell surface, while this effect was reversed by TDCA treatment (**Figure 1F**). In contrast, the surface expression of P2X7R was upregulated by Aβ ± ATP and was inhibited by TDCA treatment (**Figure 1F**; **Supplementary Figure 3**). In BV2 cells, GPCR19 (green) and P2X7R (red) showed colocalization (yellow) in resting state (**Figure 1F**). Colocalized area decreased after treatment with Aβ ± ATP, which was upregulated by TDCA treatment (**Figure 1F**).

In the frontal cortex of 5xFAD mice, GPCR19 expression was significantly increased after treatment with TDCA (1 mg/kg, i.p., q.d. for 10 weeks) compared with PBS-treated mice (**Figure 1G**; **Supplementary Figure 4**). However, the expression levels of P2X7R were significantly lower in 5xFAD-TDCA mice than in 5xFAD-PBS mice (**Figure 1G**; **Supplementary Figure 4**). Colocalization areas (yellow) of GPCR19 (green) with P2X7R (red) in the brain were significantly lower in 5xFAD-PBS mice compared with 5xFAD-TDCA mice (**Figure 1G**).

### Roles of GPCR19 for P2X7R-Mediated Ca^++^ Mobilization in Microglia

**Figure 2:**
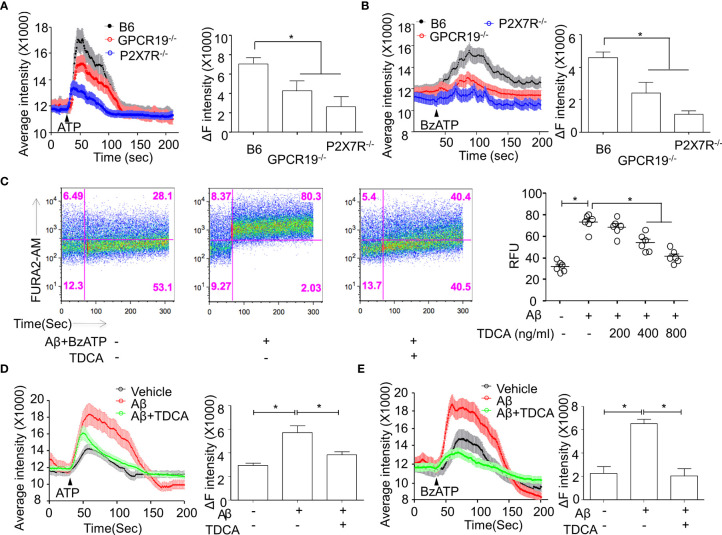
Roles of GPCR19 for P2X7R-mediated Ca^++^ mobilization in microglia. **(A, B)**, Primary microglia from B6, GPCR19^-/-^, and P2X7R^-/-^ mice (n = 6/group) were stimulated with ATP **(A)** (40 µM) or BzATP **(B)** (40 µM). Average intensity reflecting intracellular Ca^++^ mobilization (shown in the left panels) and delta intensity (max-min) after stimulation were analyzed (right panel). **(C)**, Ca^++^ mobilization of BV2 cells was measured after treatment with Aβ, BzATP (300 µM), and TDCA using flow cytometry. Representative FACS plots are shown in the left panels, and RFUs from three independent experiments are depicted in the right panel. Ca^++^ mobilization of BV2 cells in response to Aβ and TDCA in the presence of ATP **(D)** or BzATP **(E)** is depicted. The individual samples are shown with the mean ± SEM and *P < 0.05 using Student’s unpaired t-test.

Primary microglia were isolated from the brains of B6, P2X7R^-/-^, or GPCR19^-/-^ mice. In response to P2X7R agonists (ATP or BzATP), Ca^++^ mobilization was significantly decreased in the microglia from P2X7R^-/-^ or GPCR19^-/-^ mice compared with microglia from B6 mice (**Figures 2A, B**). When treated with Aβ, Ca^++^ mobilization of BV2 cells was significantly inhibited by TDCA in response to BzATP in a dose-dependent manner (**Figure 2C**; **Supplementary Figure 5**). ATP (**Figure 2D**) or BzATP (**Figure 2E**) increased Ca^++^ mobilization of BV2 microglial cells in the presence of Aβ, and Ca^++^ mobilization was significantly inhibited by TDCA (400 ng/ml).

### TDCA Suppresses N3I Activation of Microglia by Aβ ± ATP

**Figure 3:**
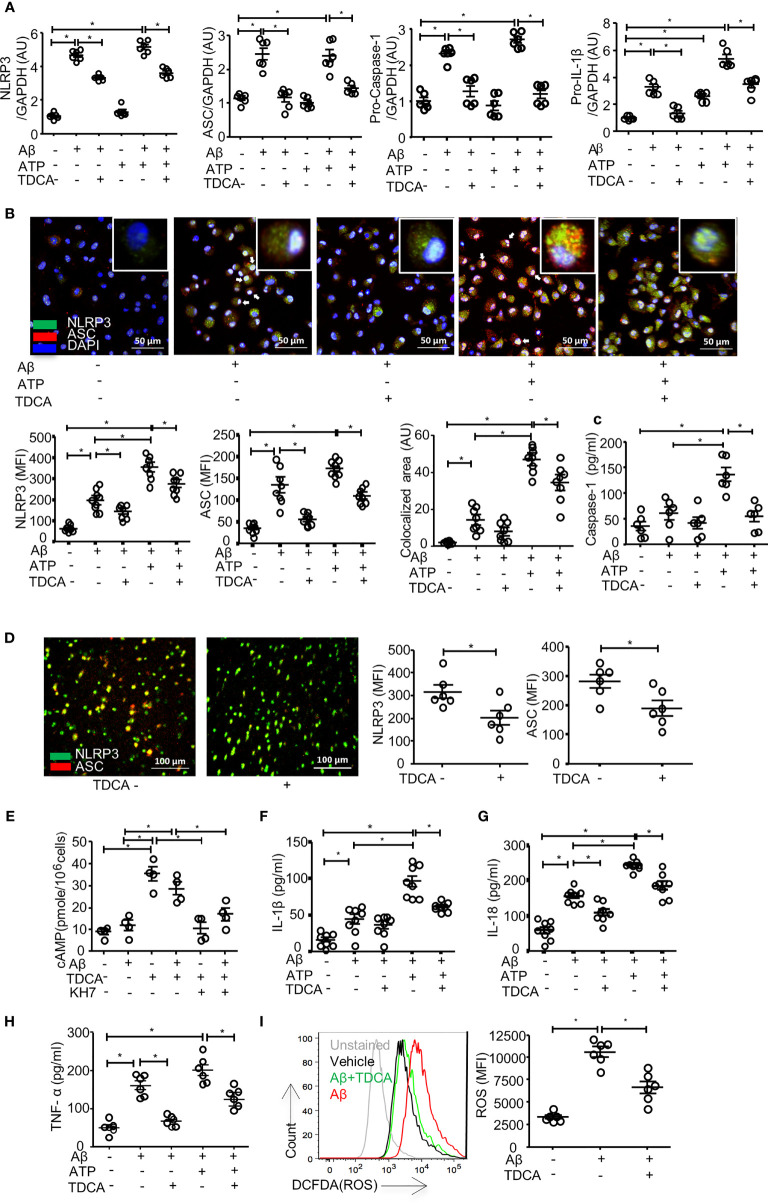
TDCA suppresses activation of the NLRP3 inflammasome and inhibits the production of IL-1β, IL-18, TNF-α, and ROS in microglia. **(A)**, Transcripts of *NLRP3, ASC, pro-caspase-1*, and *IL-1β* in BV2 cells were quantitated using qPCR after treatment with Aβ, TDCA, and ATP. Gene expression was normalized using *GAPDH.* **(B)**, The expression of NLRP3 (green) and ASC (red) in primary microglia was determined using confocal microscopy. Nuclei were stained with DAPI (blue). Colocalization of NLRP3 and ASC (yellow) was analyzed using ImageJ. **(C)**, Concentrations of caspase-1 in culture supernatants of primary microglial cells were analyzed using ELISA. **(D)**, 5xFAD mice (n = 6/group) were treated with 1 mg/kg TDCA i.p. for 10 weeks, and the expression levels of NLRP3 (green) and ASC (red) in the frontal cortex were analyzed using confocal microscopy. **(E)**, The level of cAMP in the BV2 cell lysates was measured. BV2 cells were also cultured in the presence of the adenylate cyclase inhibitor KH7 (4 µM). IL-1β **(F)**, IL-18 **(G)**, and TNF-α **(H)** in the culture supernatants of BV2 cells were analyzed using ELISA. **(I)**, ROS production in BV2 cells was quantitated using FACS after pulsing cells with DCFDA. A representative FACS plot (left panel) of three independent experiments (right panel) is depicted. The individual samples are shown with the mean ± SEM and *P < 0.05 using Student’s unpaired t-test.

It was reported that Aβ (2.5~5 µM) could induce caspase-1-mediated IL-1β secretion by microglia (48). When BV2 cells were treated with Aβ (2 µM) ± ATP (1mM), the transcript levels of NLRP3, ASC, pro-caspase-1, and proIL-1β were increased (**Figure 3A**). Although Aβ (2 µM) alone could increase these transcripts, ATP alone did not increase these transcript levels (but proIL-1β), suggesting that Aβ (2 µM) alone could activate priming phase of NLRP3 inflammasome. TDCA significantly inhibited the upregulation of these transcripts increased by Aβ ± ATP. In primary microglial cells, Aβ treatment significantly increased the expression of NLRP3 and ASC (**Figure 3B**). Treatment with Aβ + ATP further increased the expression of NLRP3. Aβ ± ATP treatment increased expression of NLRP3 and ASC in nucleus (white arrows) and in the cytosolic compartment (yellow, **Figure 3B**). TDCA treatment significantly suppressed the expression of these two molecules, as well as their colocalization (yellow dots, **Figure 3B**; **Supplementary Figure 6**). Interestingly, the levels of nuclear NLRP3 (white arrows) were significantly downregulated by TDCA treatment. Caspase-1 secretion from primary microglia treated with Aβ + ATP was inhibited by TDCA treatment (**Figure 3C**). *In vivo*, TDCA administration (1 mg/kg, i.p., q.d.) for 10 weeks decreased the expression of NLRP3 and ASC in the frontal cortex of 5xFAD mice compared with PBS-treated 5xFAD mice (**Figure 3D**; **Supplementary Figure 7**).

To elucidate how TDCA inhibits the transcription and expression of N3I components, we analyzed the GPCR19-cAMP-PKA-NF-kB axis after BV2 cells were treated with Aβ ± TDCA (39) (**Figures 3E–I**). TDCA increased cAMP production in BV2 cells irrespective of Aβ treatment (**Figure 3E**). Adenylyl cyclase inhibitor (KH7) blocked TDCA-mediated cAMP production in BV2 cells. BV2 cells increased the production and secretion of IL-1β, IL-18, and TNF-α upon treatment with Aβ ± ATP, while TDCA suppressed the production of these cytokines (**Figures 3F–H**). BV2 cells produced ROS in response to Aβ and was inhibited by TDCA treatment (**Figure 3I**).

### TDCA Decreases Apoptosis of Neurons in 5xFAD Mouse Brains

**Figure 4:**
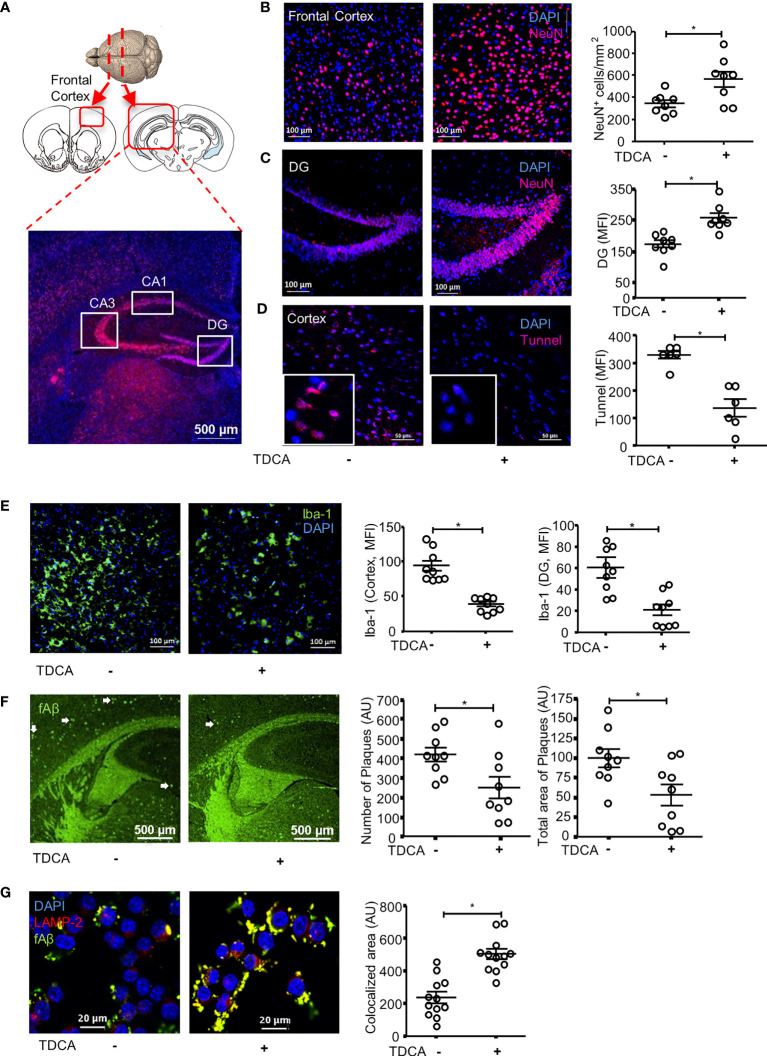
TDCA prevents apoptosis of neurons, decreases reactive microgliosis and Aβ plaques in the brain. **(A)**, 5xFAD mice (n = 8/group) were treated with 1 mg/kg TDCA i.p. for 10 weeks. NeuN^+^ cells in the frontal cortex and hippocampus were analyzed using confocal microscopy. Paraffin sections were made from the frontal cortex **(B)** and DG of the hippocampus **(C)**. A set of representative images is depicted in the left panel, and the number of NeuN^+^ cells **(B)** or MFI ± SEM **(C)** is shown in the right panel. **(D)**, Apoptotic cells in the brain were quantitated after TUNEL staining. The nuclei were stained with DAPI (blue). **(E)**, Reactive microgliosis in the frontal cortex and DG of 5xFAD mice analyzed with confocal microscopy after staining with anti-Iba-1 Ab (left panel). **(F)**, Aβ plaques in the brain sections were stained with thioflavin-S (green dots indicated with white arrows). The number of plaques and total area of plaques were quantitated using ImageJ. **(G)**, The effects of TDCA (400 ng/ml) on phagocytosis of fAβ by BV2 cells were tested. Colocalization (yellow) of fAβ (green) with phagosomes were determined using confocal microscopy after staining with anti-LAMP2^+^Ab (red). The area of colocalization (yellow) were quantified using ImageJ from four independent experiments (triplicate, right panel). The individual samples are shown with the mean ± SEM and *P < 0.05 using Student’s unpaired t-test.

NeuN^+^ cells in the brains of 5xFAD mice were stained after treatment with TDCA for 10 weeks (1 mg/kg, i.p., q.d., **Figure 4**). The frontal cortexes and hippocampi (CA1, CA3, and DG) were observed using confocal microscopy (**Figure 4A**). Number of NeuN^+^ cells in the frontal cortex were significantly higher in 5xFAD mice treated with TDCA than in those of 5xFAD mice treated with PBS (**Figure 4B**; **Supplementary Figure 8A**). The mean fluorescent intensity (MFI) of the NeuN^+^ area in the hippocampi of 5xFAD mice was significantly higher in the TDCA group than in the PBS group (**Figure 4C**; **Supplementary Figures 8B-D**). MFI of TUNEL+ apoptotic cells in the cortex were significantly higher in 5xFAD mice treated with PBS than in 5xFAD mice treated with TDCA (**Figure 4D**).

### TDCA Decreases Gliosis and Aβ Plaques in the Brains of 5xFAD Mice

After i.p. administration of TDCA for 10 weeks, the numbers of Iba-1^+^ reactive microglia in the frontal cortex (**Figure 4E**; **Supplementary Figure 9A**) and in the dentate gyrus (DG) (**Supplementary Figure 9B**) of the hippocampus decreased. The number of GFAP^+^ reactive astrocytes also significantly decreased in the frontal cortex (**Supplementary Figure 10A**), DG (**Supplementary Figure 10B**), and CA3 regions (**Supplementary Figure 10C**) of the hippocampus in 5xFAD mice upon treatment with TDCA. The numbers of Aβ plaques and the total area of plaques (white arrows) in the frontal cortex and in the hippocampus of 5xFAD mice decreased after TDCA treatment (**Figure 4F**). *In vitro*, TDCA treatment increased the phagocytosis of Aβ by BV2 cells (**Figure 4G**). The phagocytosed fluorescent Aβ oligomers (fAβ) colocalized with the LAMP-2 phagosomes marker in BV2 cells (**Figure 4G**; **Supplementary Figure 11**), suggesting fAβ oligomers are in the phagosomes, not on the cell surface.

### Proteogenomic Analysis of Brain Tissues of 5xFAD Mice Treated With TDCA

**Figure 5:**
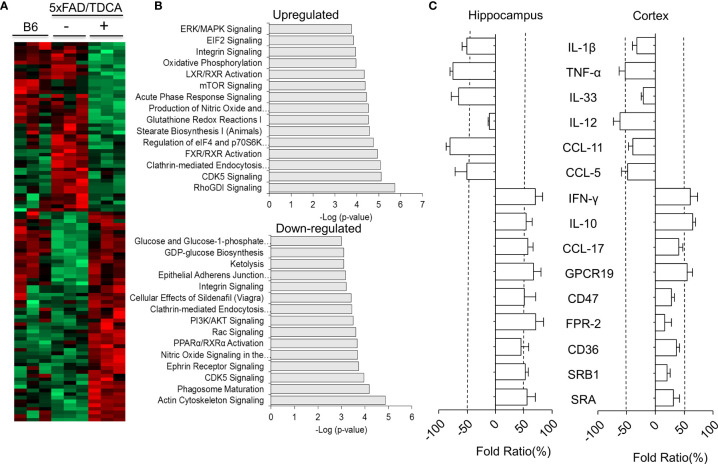
Proteomic analyses of mouse brains. B6 or 5xFAD (n = 3/group) mice were treated with TDCA (1 mg/kg i.p.) for 10 weeks. **(A)**, Heatmap showing the differential expression of proteomes in the brain. Each row represents a protein, and each column represents an individual mouse. **(B)**, The top 15 canonical pathways “upregulated” or “downregulated” by treatment with TDCA are shown. **(C)**, Cytokines, chemokines, immune checkpoint molecules, and scavenger receptors expressed in the hippocampus and cortex were determined by qPCR based on proteomic profiling. Fold ratios denote the ratio between Ct_TDCA_ and Ct_vehicle._ Fold ratios less than zero indicate that the expression was inhibited upon TDCA treatment.

After administration of TDCA (1 mg/kg, i.p., q.d.) for 10 weeks, the proteomes of brain tissues from 5xFAD mice were analyzed (**Figures 5A, B**). Total 3,259 unique proteins were identified at a protein threshold of a 1.0% false discovery rate (FDR). Among these proteins, 460 proteins showing peptide spectral counts in more than two assays from triplicate assays, with a fold change of more than 2 between PBS- and TDCA-treated groups, are depicted on the heatmap, plotted with the Perseus software platform (http://www.perseus-framework.org). Proteomic analysis indicated that 56 proteins exhibited more than 2-fold changes in the 95% confidence interval (**Figure 5A**), demonstrating two distinct proteome clusters that were upregulated in TDCA-treated groups and downregulated in PBS-treated groups, or vice versa. The functions of these proteins were further analyzed based on QIAGEN’s IPA database (**Figure 5B**). Notably, a canonical pathway, regulation of eIF4 and p70S6K, was enriched by TDCA treatment, suggesting that TDCA plays critical roles in translational regulation followed by calcium signaling, which could exert allosteric regulatory effects on many enzymes and proteins. Based on proteomic analysis, we further analyzed the transcript levels of several proinflammatory cytokines in both the hippocampi and cortices of 5xFAD mice after TDCA treatment for 10 weeks. TDCA downregulated IL-1β, TNF-α, IL-33, IL-12, CCL-11, and CCL-5 transcripts. In contrast, the TDCA treatment increased the transcript levels of IFN-γ, IL-10, CCL-17, GPCR19, CD47, FPR-2, CD36, SRB1, and SRA in the brains of 5xFAD mice (**Figure 5C**).

### TDCA Improves the Spatial Learning and Memory of 5xFAD Mice

**Figure 6:**
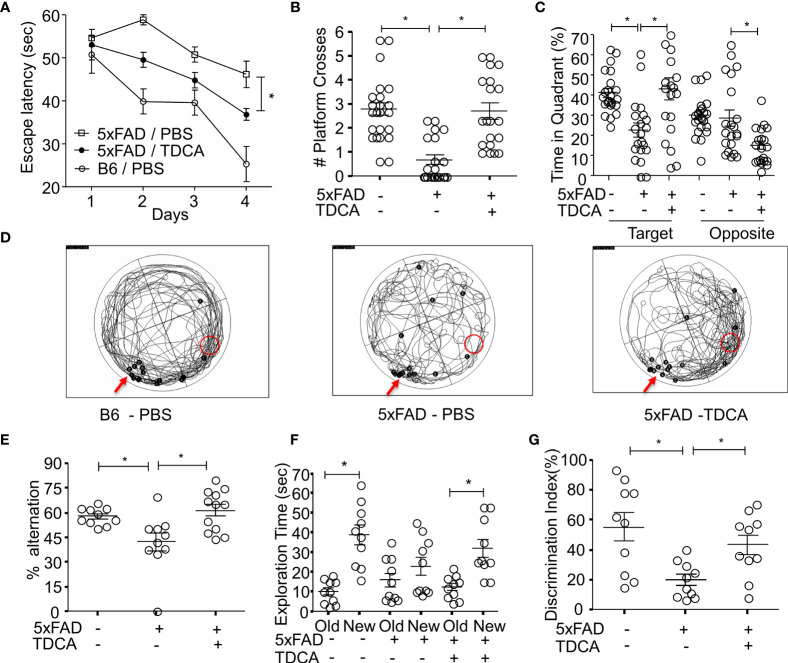
TDCA improves spatial learning and memory of the 5xFAD mouse. The learning and memory of 5xFAD and B6 (5xFAD^-^) mice were tested after treatment with TDCA 1 mg/kg i.p. for 10 weeks (n = 20~22/group). The time to reach the hidden platform (escape latency) **(A)** and the number of platform crosses **(B)** were measured using the MWM test. The time spent in the target quadrant where a platform is located, and the opposite quadrant **(C)** were calculated from the MWM test. **(D)**, Trajectory maps show the escape route of all mice in the MWM test in a set of experiments. The red arrows and red circles indicate release points and positions of the platform, respectively. Black dots indicate the beginning **(B)** and end **(E)** points for individual mice. **(E)**, Alternation percentages of mice (n = 9~12) in the Y-maze test were calculated by 100 × # spontaneous alternation/# of total arm entry. **(F)**, Total exploration time for each object of individual mice in the NOR test is depicted. **(G)**, Discrimination index (%) = 100 × time spent exploring the novel object/exploration time for both the novel and old objects for each mouse (n = 10). The individual samples are shown with the mean ± SEM and *P <0.05 using Student’s unpaired t-test.

B6 and 5xFAD mice were administered with PBS or TDCA for 10 weeks (1 mg/kg, i.p., q.d.), and spatial learning and memory were assessed using the Morris water maze (MWM) test (**Supplementary Figure 12A**). There were no significant changes in body weight after TDCA treatment for 10 weeks (**Supplementary Figure 12B**). Escape latency decreased over the 4-day training period in all three groups. Mice in the TDCA group showed a significant reduction in time latency to reach the platform (**Figure 6A**), which was comparable to that of B6 mice. In the probe test on day 5, TDCA-treated 5xFAD mice showed increased numbers of platform crossings (**Figure 6B**). TDCA-treated 5xFAD mice spent more time on the platform than the PBS group (**Supplementary Figure 12C**). TDCA-treated 5xFAD mice remained for longer periods of time in the target quadrant than in the opposite quadrant compared with PBS-treated 5xFAD mice (**Figure 6C**). Times in the target quadrant and on the platform of 5xFAD-TDCA group were comparable to those of B6 mice. The movement of 5xFAD mice treated with PBS were relatively random and disorganized compared with those of B6 mice and TDCA-treated-5xFAD mice (**Figure 6D**).

Spatial learning and memory were also assessed using a Y-maze test and novel object recognition test (NOR). In the Y-maze test, the alternation percentage of the mice in the 5xFAD-PBS group was significantly lower than that of mice in the B6-PBS group and 5xFAD-TDCA group (**Figure 6E**). In the NOR test, exploration time to a new object compared to an old object was significantly higher in B6-PBS and 5xFAD-TDCA mice (**Figure 6F**). 5xFAD-PBS mice did not exhibit a difference in exploration time between old and new objects. The discrimination index for the mice in the 5xFAD-TDCA group was significantly higher than that for mice in the 5xFAD-PBS group (**Figure 6G**).

## Discussion

Senile Aβ plaques in the human brain incur microgliosis, which is responsible for neuroinflammatory cascades. These factors cause memory and cognitive impairment, eventually progressing to dementia (6). Reactive microgliosis accompanied by neuronal damage aggravates AD (5). ATP released from damaged neurons can activate P2X7R in AD (49). Aβ-mediated activation of N3I exacerbates the pathogenesis of AD by inducing neuroinflammation (15). However, interventions using N3I activation to suppress neuroinflammation in AD are still not successful. Therefore, many resources have been proposed to halt the progression of Aβ-mediated N3I activation, which might delay neuronal loss due to the neuroinflammatory cascade. Focusing on CNS inflammation would be a major gateway to control AD-related dementia. Based on our previous studies on toxic dosage (50, 51) and effective dosage of TDCA in sepsis models (52), we used TDAC in 1 mg/kg, i.p. (10 weeks) for this study. We found that administration of a GPCR19 agonist, TDCA, significantly improved the learning and memory of 5xFAD mice (**Figure 6**).

The possible mode of action of TDCA could be explained in three ways. First, TDCA might suppress the priming phase of N3I activation (transcription of NLRP3, ASC, and pro-caspase-1; **Figures 3A, B**) and the activation phase of N3I (production of mature IL-1β and IL-18 by NLRP3-ASC oligomerization; **Figures 3F, G**) in response to Aβ and ATP. Second, TDCA might inhibit the production of crucial proinflammatory mediators in microglia, such as ROS and TNF-α, independent of N3I activation (**Figures 3H, I**). Finally, TDCA may augment the clearance of Aβ by enhancing phagocytosis and by suppressing P2X7R expression (**Figures 4F, G**). Overall, TDCA reduces neuroinflammation and prevents neuronal apoptosis, which delays the impairment of spatial learning and memory (**Figure 6**).

TDCA regulates N3I activation by altering the functions and expression of P2X7R after binding with GPCR19 (**Figures 1F, G**). The P2X7R on microglia is crucial in Ca^++^ mobilization, which initiates N3I activation (53). Many glial functions such as the production of cytokines and chemokines are mediated by Ca^++^ (30, 54).

Upon stimulation with P2X7R agonists (ATP or BzATP), GPCR19^-/-^ microglia were unable to mobilize cytosolic Ca^++^ as much as WT microglia (**Figures 2A, B**), suggesting that GPCR19 might be necessary in Ca^++^ mobilization in response to P2X7R agonists within 100 sec. ATP activates channel function of P2X7R on the cell membrane, encouraging the exchange of cytosolic and extracellular cations (55). The crystal structure of the P2X7R receptor displays clear differences between the open and closed pore states (56). However, no studies have been reported on the role of GPCR19 in this process until now. In this study, several findings suggest a biphasic role of GPCR19 in regulating P2X7R. In the immediate phase in response to proinflammatory Aβ and/or ATP (50–150 sec), TDCA may regulate the function of P2X7R *via* allosteric modulation of GPCR19, which interferes with the GPCR19-P2X7R interaction. GPCR19 and P2X7R are colocalized on the cell membranes of resting microglia (**Figure 1**), and Ca^++^ is mobilized within 100 sec in response to ATP. A GPCR19 agonist, TDCA, inhibited Ca^++^ mobilization when microglia were stimulated with an Aβ + P2X7R agonist (ATP or BzATP) within 100 sec. This further supports the idea that GPCR19 might be necessary for the activation of P2X7R, and TDCA interferes with the interaction of these two molecules (**Figures 2D, E**). This finding suggests that binding of TDCA with GPCR19 might cis-regulate the opening the pore of P2X7R. The binding of TDCA with GPCR19 might alter tertiary structure of GPCR19, which is necessary for opening the pore of P2X7R in response to its ligands. In a delayed phase (1 h), TDCA suppresses P2X7R expression and increases the expression of GPCR19 on the cell membrane in response to Aβ and/or ATP (**Figures 1F, G**), suggesting plausible trans-regulation of P2X7R expression by the GPCR19-mediated signaling cascade. In summary, the TDCA-GPCR19 complex might transmit signals necessary for inhibiting P2X7R expression on the membrane within an hour (delayed response) or might alter GPCR19 structures necessary for the Ca^++^ current incurred by P2X7R activation within a couple of seconds (immediate response). It was reported that Aβ (2.5~5 μM) could induce caspase-1-mediated IL-1β secretion by microglia (48). We also observed that Aβ alone (2 μM) increased the production of IL-1β and IL-18 (**Figures 3F, G**). However, the production of IL-1β and IL-18 by Aβ (2 μM) +ATP (1 mM) was significantly higher than that by Aβ alone (2 μM). Aβ alone (2 μM) did not increase cytoplasmic Ca^++^ without ATP or BzATP (**Figure 2D**). These findings suggest that Aβ alone (2 μM) is not sufficient to activate P2X7R-mediated activation of the NLRP3 inflammasome in our setting. The membrane pores were formed by more than 20 μM Aβ (57). Lučka Bibič and Leanne Stokes used higher concentration of Aβ (30~100 μM) to increase cytoplasmic Ca^++^, which was independent of P2X7R (58). Taken together, Aβ (2 µM)-induced caspase-1 activation might be mainly due to K^+^ efflux/Ca^++^ induction, not by pore formation in our study. In the brains of 9-month-old 5xFAD mice, the expression of GPCR19 was significantly lower, and the expression of P2X7R was significantly higher than the levels observed in the brains of 3-month-old 5xFAD mice or B6 mice (**Figure 1D**). The expression levels of GPCR19 and P2X7R on microglia were reciprocally regulated after stimulation with Aβ ± ATP *in vitro*, which was reversed by TDCA treatment (**Figure 1F**). Taken together, these findings suggest that neuroinflammation induced by Aβ ± ATP might be responsible for the downregulation of anti-inflammatory GPCR19 and the upregulation of proinflammatory P2X7R upon aging. The relative expression levels of GPCR19 and P2X7R on microglia might be crucial biomarkers indicating the severity of neuroinflammation in AD patients.

Proteomic analysis of brain lysates exhibited global editing of the brain proteome, which leads to an anti-inflammatory microenvironment in the AD brain (**Figure 5A**). For example, upregulation of clathrin-mediated endocytosis, FXR pathway, mTOR signaling, and acute phase response signaling were observed after treatment with TDCA, in addition to downregulation of NO signaling and PI3K/AKT signaling (**Figure 5B**).

Various bile acids are GPCR19 agonists and can reduce inflammation in the brain (44). However, many studies have been carried out with bile acids at concentrations that are unobtainable under pharmacological or pathological conditions *in vivo (* 59). In addition, efficacy of INT-777 was tested in mice after intracerebroventricular injection (60). Thus, the exact role of bile acids in modulating brain inflammation in AD could not be concluded. In this study, we showed that TDCA, one of the bile acids interacting with GPCR19 (61), could suppress brain inflammation in 5xFAD mice by inhibiting the P2X7R-N3I axis. Furthermore, i.p. injection of TDCA also improved spatial learning and memory. Considering the safety profiles of TDCA after i.v. administration (51), TDCA may provide a viable option for AD patients to ameliorate neuroinflammation, which in turn might delay the progression of AD.

## Materials and Methods

### Human Brain Samples

A total of eight postmortem brain samples were obtained (**Supplementary Table 1**); five from individuals with clinically and neuropathologically diagnosed AD (Braak stages: V–VI) and three from nondemented controls. Brain sections were provided by the Korea Brain Bank Network (KBBN) operated through the National Brain Bank Project funded by the Ministry of Science and ICT. The project was approved by the Scientific Committee of the Brain Bank.

### Reagents

TDCA was purchased from New Zealand Pharmaceuticals Ltd. (Palmerston North, New Zealand). KH7 (Ann Arbor, Michigan, USA) was dissolved in DMSO. ATP and BzATP (Sigma–Aldrich, St. Louis, MO, USA) were dissolved in PBS.

### Preparation of Aβ_1-42_ Oligomer

A lyophilized 0.1 mg vial of amyloid β protein fragments 1-42 (Aβ; Sigma–Aldrich) was dissolved in 50 µl DMSO (Sigma–Aldrich) for 1 h at room temperature with continuous rotation. Oligomeric Aβ_1-42_ was prepared by diluting the dissolved Aβ to 100 µM using DMEM/F12 media. The resulting solution was then incubated for 24 h at 4°C with continuous rotation.

### Isolation of Primary Microglia and Culture

Primary microglia were obtained from the cerebral cortices and hippocampi of male and female 1- to 2-day-old B6 mice or adult B6, GPCR19^-/-^, and P2X7R^-/-^ mice (Jackson Laboratory, USA) using a mouse brain dissociation kit (Miltenyi Biotec GmbH, Bergisch, Gladbach, Germany). The tissue was cut into fragments using a scalpel blade (Medicom, Los Angeles, CA, USA) and then homogenized using a gentleMACS(TM) C tube and gentleMACSTM Octo Dissociator (Miltenyi Biotec GmbH). Single-cell suspensions were obtained after passing the tissue homogenates through MACSR Smart Strainers (70 µm; Miltenyi Biotec GmbH). The myelin was depleted using a debris removal solution supplied with the kit. Microglial cells were isolated using CD11b MicroBeads, LS Columns, and a MACS multistand system (all obtained from Miltenyi Biotec GmbH). Isolated microglia were washed with PB buffer (0.5% FBS in PBS) and cultured using DMEM-F12 media with 20% FBS and 10 ng/ml M-CSF (PeproTech, Rocky Hill, NJ) in a 95% air and 5% CO2 atmosphere at 37°C.

For inflammasome activation, primary microglial cells from 1- to 2-day-old B6 mice were seeded on 12-mm microscope cover glasses (Deckglaser, Luda-Konlgshofen, Germany) in 24-well PDL-coated cell culture plates (Corning, Wujiang, Jiangsu, China) at 7.5 x 10^4^ cells/ml per well for 24 h, serum starved for 10 h and treated with Aβ (2 µM), TDCA (400 ng/ml) for 24 h, and ATP (1 mM) for the final hour.

### Cell Culture

BV2 cells (Murin microglial cell line) were maintained in a 95% air and 5% CO_2_ atmosphere at 37°C in DMEM (Dulbecco’s modified Eagle’s medium complete media; Invitrogen, Carlsbad, CA, USA) containing 10% FBS (Fetal Bovine Serum; Invitrogen) and 1% penicillin and streptomycin (Invitrogen). BV2 cells were seeded in 6-well (1.5 x 10^5^ cells/2 ml), 12-well (1 x 10^5^ cells/ml), or 24-well (5 x 10^4^ cells/0.5 ml) PDL-coated cell culture plates for 12 h using DMEM complete media. Cells were serum starved before treatment for 10 h using DMEM/F12 media (Thermo Fisher Scientific, Waltham, MA, USA). Cells were treated with Aβ (2 µM), TDCA (400 ng/ml), and ATP (1 mM) for 1 h in the case of GPCR-19–P2X7R interaction. For inflammasome activation and inhibitor (KH7) assays, cells were treated with Aβ (2 µM), KH7 (4 µM), TDCA (400 ng/ml) for 24 h, and ATP (1 mM) for the final hour. DMEM/F12 medium was used unless stated otherwise. The cell-lysed supernatants were used to check the concentrations of cAMP, and cell culture supernatants were used to analyze the concentrations of IL-1β, IL-18, TNF-α, and caspase-1 using ELISA.

### Measurement of Microglial Ca^++^ Response

Adult mouse microglial cells isolated from adult B6, GPCR19^-/-^, and P2X7R^-/-^ mice were seeded on 25-mm cover glasses (Deckglaser) in a 6-well plate. After 48 h of culture with DMEM/F12 medium containing 20% FBS and 10 ng/ml M-CSF in a 95% air and 5% CO_2_ atmosphere at 37°C, cells with glass coverslips were transferred and loaded with 2 µM Fluo-4/AM for 30 min at 37°C in a physiological external solution consisting of 138 mM NaCl, 5.6 mM KCl, 0.5 mM MgCl2, 2 mM CaCl2, 10 mM HEPES, and 10 mM glucose (pH 7.4). After loading, cells on the coverslips were transferred to an open perfusion chamber, and fluorescence was measured at 494/506 nm using a fluorescence microscope (Nikon, Tokyo, Japan). The microscope was equipped with an LED lamp (Andover, UK), integrated shutter, and cooled EM-CCD camera. The camera and shutter were controlled using MetaMorph software (Molecular Devices, Foster City, CA). Single cells were defined as regions of interest (ROIs). Sixteen-bit grayscale images with a binning of 1 x 1 were captured every 1 s with a ranging exposure time. Data were processed using OriginPro 8 software (OriginLab) and merged from three independent experiments.

BV2 cells were seeded on 25-mm cover glasses in a 6-well plate. After starvation, cells were treated with Aβ (2 µM) with or without TDCA (400 ng/ml) for 24 h. Cells with glass coverslips were transferred and loaded with 2 µM Fluo-4/AM for 30 min at 37°C in the physiological external solution mentioned above. After loading, the cells were transferred to an open perfusion chamber, and fluorescence was measured at 494/506 nm as previously described.

BV2 cell Ca^++^ sensing was measured using a BD calcium assay kit (BD Bioscience, San Diego, CA, USA) according to the manufacturer’s protocol. In a 6-well plate, 1.5 x 10^5^ cells/2 ml media were treated with Aβ with or without TDCA for 24 h after serum starvation. Cells were harvested from the cell culture plate in a FACS tube, washed with complete RPMI media, loaded with loading dye, and incubated for 1 h at 37°C in a 5% CO_2_ atmosphere. Cells were acquired for 1 min for basal signaling, incubated with BzATP (300 µM) for 2 min, and recorded for an additional 4 min using flow cytometry (LSRFortessa, BD Biosciences, San Jose, CA, USA). Relative fluorescent units (RFUs) were calculated using FlowJo version 9.0 (Treestar, Ashland, OR, USA).

### Immunoblot

Cortices were harvested from 3 sets of B6 (3-month-old) and 5xFAD (3-, 4-, 6-, and 9-month-old) mice, lysed with RIPA buffer (Thermo Fisher Scientific, Meridian Road, Rockford, USA) supplemented with phosphatase inhibitor cocktail 2 (Sigma–Aldrich), and centrifuged to remove cell debris. The concentrations of the prepared protein samples were determined using a BCA kit (Thermo Fisher Scientific). Protein samples were separated by electrophoresis on 10% sodium dodecyl sulfate–polyacrylamide gels and then transferred electrophoretically to immobilon, polyvinylidene-difluoride membranes (Merck, Millipore, Billerica, MA, USA). The membranes were incubated at 4°C overnight with anti-GPCR19 polyclonal antibody (Novus Biologicals, Littleton, CO, USA), P2X7R monoclonal antibody (Clone 1F11, Biolegend, San Diego, CA, USA), and anti‐β‐actin (Sigma–Aldrich). The following day, the membranes were washed and then incubated with horseradish peroxidase‐labeled anti‐rabbit or anti‐mouse secondary antibodies for 1 h at room temperature. Subsequently, membrane‐bound horseradish peroxidase‐labeled antibodies were detected using an enhanced chemiluminescence detection system, including the Pierce ECL Western Blotting Substrate kit (Thermo Fisher Scientific). Densitometric quantification of the bands was conducted using ImageJ software (Rasband, W.S., NIH, Maryland, USA). Protein levels were normalized to β‐actin for quantification.

### Immunocytochemistry

BV2 cells or primary microglial cells isolated from adult mouse brains were seeded on 12-mm microscope cover glasses (Deckglaser) in 24-well plates. Cells were stained with anti-GPCR19 polyclonal antibody (Novus Biologicals) and P2X7R (Clone 1F11, Biolegend) at 4°C overnight, followed by staining with goat anti-rabbit IgG, Alexa Fluor 488 (Thermo Fisher Scientific), and goat anti-rat IgG, Alexa Fluor 546 (Invitrogen) for 1 h at RT.

To stain for inflammatory components, primary microglial cells isolated from the brains of 1- to 2-day-old B6 mice were seeded on 12-mm microscope cover glasses in 24-well plates and treated with Aβ (2 µM), TDCA (400 ng/ml) for 24 h, and ATP (1 mM) for the final hour. The cells were permeabilized and stained with anti-NLRP3 polyclonal antibody (Abcam, Cambridge, United Kingdom) and anti-ASC antibody (Clone B-3, Santa Cruz Biotechnology, Inc. Dallas, Texas, USA) at 4°C overnight, followed by staining with secondary polyclonal antibodies, Alexa Flour 488-labeled goat anti-rabbit IgG, or Alexa Flour 532-labeled goat anti-mouse IgG (Invitrogen) for 1 h at RT.

After rinsing with secondary polyclonal antibodies, cover glasses were placed on DAPI mounting solution (Vector laboratories, Burlingame, CA, USA) on glass slides. Fluorescence imaging was performed using a Confocal Microscope A1 (Nikon, ECLIPSE Ti, New York, USA). The colocalization percentage of NLRP3-ASC was measured using ImageJ software after setting the color threshold caliper to only yellow. NIS-Elements.AR. Ink (version 4.2, Nikon) was used to measure the mean fluorescence intensity (MFI) of the ROIs.

### Quantitative RT–PCR

Tissue or cells were harvested after treatment, and total RNA was isolated using an RNeasy Plus Mini kit (QIAGEN, Hilden, Germany). cDNA was prepared from 1 µg of total RNA using a Maxime RT PreMix kit (iNtRON Biotechnology, Gyeonggi-do, South Korea). Thereafter, real-time quantitative PCR (qPCR) was performed using SYBR Green Fast mix (Applied Biosystems, Woolston, Warrington, UK) and primers specific to target genes (**Supplementary Table 2**) in the StepOnePlus™ Real-Time PCR system (Applied Biosystems, Marsiling Industrial Estate Road, Singapore). The expression levels of target mRNAs were analyzed using the ddCt method and were normalized to the expression levels of mouse *GAPDH*, a housekeeping gene used as an endogenous control. All fold changes are expressed relative to the control group.

### ELISA

Cell-secreted cytokines were measured from the cell culture supernatant using commercially available ELISA kits for mouse TNF-α, IL-1β/IL-1F2 (R&D Systems, Minneapolis, MN, USA), IL-18 (MBL, Naka-Ku, Nagoya Aichi, Japan), and Caspase-1 (Novus Biologicals) according to the manufacturer’s protocols. BV2 cells were cultured in the presence of Aβ (2 µM), TDCA (400 ng/ml), or KH7 (4 μM) for 24 h. Cells were harvested and lysed with 0.1 M HCl to measure intracellular cAMP using a cAMP assay kit (Abcam) according to the manufacturer’s protocol.

### Measurement of ROS Production

BV2 cells were harvested in a FACS tube after treatment with Aβ (1~4 µM) and TDCA (400 ng/ml) for 24 h. Cells were washed with DPBS and incubated with 5 μM 2′-7′-dichlorofluorescin diacetate (Invitrogen, Eugene, Oregon, US) in DMEM containing 1% penicillin and streptomycin for 30 min at room temperature in the dark. Samples were washed in DPBS, suspended in FACS buffer containing DAPI (0.3 µg/ml), immediately acquired and analyzed using a BD LSR Fortessa flow cytometer (BD Bioscience, San Jose, CA, USA) and FlowJo v9 software.

### Phagocytosis of fAβ Oligomer

Primary microglia isolated from 1- to 2-day-old B6 mice were cultured, pretreated with TDCA (400 ng/ml) for 12 h, and then incubated with fAβ (1–42) oligomer (green) for 3 h. After incubation with fAβ, the cells were washed with ice-cold PBS and fixed with 4% paraformaldehyde (PFA). After blocking, the cells were incubated at 4°C overnight with a primary antibody against P2X7R (BioLegend) or control IgG. The cells were then rinsed and incubated with goat anti-mouse IgG (H+L) secondary antibody (Invitrogen) for 1 h at room temperature in the dark. After rinsing, cover glasses were placed on the DAPI mounting solution on the glass slides. Fluorescence imaging was performed using a confocal microscope A1. NIS-Elements.AR. Ink (version 4.2, Nikon) was used to measure the MFI of ROIs.

BV2 cells were seeded on microscope cover glasses in 24-well plates for confocal microscopy. Cells were treated with or without TDCA for 12 h. HilyteFluor™ 488-labeled Aβ (1-42; AnaSpec, Freemont, CA, USA) was used for fAβ oligomers, as mentioned above. After pretreatment with TDCA, the cells were incubated with fAβ oligomers for 3 h, fixed with 4% paraformaldehyde (PFA), and permeabilized with 0.3% Triton X-100. After blocking, the cells were incubated at 4°C overnight with primary antibody for LAMP2 (Clone M3/84, BD Pharmingen Inc., San Jose, CA, USA) or control rat IgG. After rinsing, the cells were incubated with goat anti-rat IgG, Alexa Fluor 546 (Invitrogen) for 1 h at room temperature in the dark. After rinsing, cover glasses were placed on the DAPI mounting solution on the glass slides. Fluorescence imaging was performed using a confocal microscope A1. The colocalization percentage of fAβ-LAMP2 was measured using ImageJ software after setting the color threshold caliper to only yellow.

### Animals

The 5xFAD mice co-overexpress high levels of APP with three FAD mutations (Swedish (K670N/M671L), Florida (I716V), and London (V717I)) and high levels of presenilin 1 (PSEN1) with two FAD mutations (M146L, L286V), which are specifically overexpressed in the brain and regulated by the neural-specific Thy1 promoter (62). 5xFAD mice were maintained by breeding male 5xFAD mice with female B6 mice. The SJL F1 hybrid was produced by an SJL male and a B6 female. PCR was performed to genotype the mice. 5xFAD or SJL mice were kindly provided by Professor Mook-Jung, In-hee or Professor Sung, Jung-Joon, respectively, of Seoul National University. GPCR19^-/-^ mice (C57BL/6-Gpbar1^tm1(KOMP)Vlcg^ mice) and P2X7R^-/-^ mice (C57BL/6. 129P2-P2rx7^tm1Gab/^J) used in this study were purchased from Jackson Laboratory. All animal experiments were approved by the institutional animal care and use committees (IACUC) of Seoul National University (SNU-170517-25) and performed in accordance with animal ethics regulations. Mice were maintained in specific pathogen-free conditions at the animal facility of the Wide River Institute of Immunology.

### Morris Water Maze Test

Male and female 5xFAD mice (8 to 10 weeks old) were injected intraperitoneally (i.p.) with either 1 mg/kg TDCA or PBS five times/week for 10 weeks. Age- and gender-matched nontransgenic littermates were used as a control group. Behavioral tests were performed after treatment, and then mice were sacrificed for further experimentation.

The maze was composed of a circular pool (1.5 m in diameter, 80 cm in height) with spatial cues at three different locations. Before testing, the pool was filled with opaque water adjusted to 20 ± 1 °CC. On the first day, mice were allowed to freely swim in the water for 60 sec to find the escape platform located in one quadrant of the pool. When the mice failed to find the platform, they were guided to the platform. Once on the platform, mice were allowed to remain there for 30 sec. From the next day for 4 consecutive days, the same procedure was repeated from three different starting points to train the mouse, and the time to reach the platform was recorded every day. After the 4-day training period, the probe test was performed in the same manner but without the platform. Each mouse was allowed to swim from one starting point for 60 sec, which was recorded using a video camera. The video was analyzed for the movement of mice in the water using tracking software (SMART3.0, Panlab Harvard Apparatus, Barcelona, Spain) to count the number of crossings and the time on the platform and to measure the time spent in each quadrant of the pool.

### Y-Maze Test

Mouse functional behavior tests were performed on TDCA- or PBS-treated 5xFAD mice and WT (B6) control mice. The Y-maze test was assessed over the course of four days. On the first 2 days, individual mice were habituated to the task room and experimenter for 5 min. On the third day, after the task room and experimenter habituation, mice were allowed to habituate to the Y-maze for less than 1 min. On the last day, mice entered the middle of the Y-maze and were allowed to move freely within the maze for 8 min. Each mouse movement was recorded using a video camera. The video was analyzed for all mouse entries regarding limbs that pass through each half arm of the maze. Total arm entries and the percentage of alteration were counted for each mouse and compared between groups of mice.

### Novel Object Recognition Test (NORT)

NORT was assessed over the course of 4 days. The apparatus consisted of a white acrylic box (350 mm x 450 mm x 250 mm). The basement of the box was divided into six equal rectangles. On the first 2 days, each mouse was habituated to the box for 10 min. On the third day, two similar cylindrical objects were fixed in the box, and mice were allowed to explore the objects freely for 10 min. On the last day, one cylindrical object was replaced by a similarly sized object, and the mouse explored this for 5 min. Exploration of two cylindrical objects on the third day and exploration of the novel object and cylindrical object on day 4 were recorded for each mouse using a video camera. The total time and frequency of novel and old object exploration were counted for each mouse using video footage. The percent discrimination index of the novel object was calculated from the exploration time of novel and old objects.

### Immunohistochemistry

Human samples of brain paraffin tissue sections (4 µM) from KBBN were used for cell surface GPCR19 and P2X7R expression. Tissue sections were deparaffinized in xylene and rehydrated in a graded ethanol series (100%, 90%, 80%, and 70%). Antigen unmasking was performed by heating the brain sections in citrate-based buffer (pH 6, Vector laboratories, Burlingame, CA, USA). The sections were blocked with a blocking solution of 10% normal donkey serum (Thermo Fisher Scientific) and 1% BSA in PBS for 1 h. Tissue samples were incubated overnight at 4°C with GPCR19 (Novus Biologicals) and P2X7R (Novus Biologicals) primary antibodies. Slices were subsequently incubated for 1 h at room temperature with Donkey anti-Rabbit Alexa 488 (Invitrogen) or Donkey anti-Goat Alexa 555-conjugated (Invitrogen) IgG secondary antibodies and then counterstained with DAPI for 10 min.

TDCA- or PBS-treated 5xFAD or B6 mice were anesthetized and perfused with ice-cold PBS. Brains were harvested and maintained in 10% neutral buffer formalin (Sigma–Aldrich) for 24 h at 4°C and then embedded in paraffin (Lecia, Illinois, USA). The paraffin-embedded brains were cut (3 μM) using a microtome (Thermo Fisher Scientific), deparaffinized in xylene, and rehydrated in a graded ethanol series (100%, 90%, 80%, and 70%). Antigen unmasking was performed by heating the brain sections in citrate-based buffer (pH 6, Vector laboratories, Burlingame, CA, USA). The sections were incubated in 0.3% Triton X-100 for 30 min at room temperature for intracellular staining and then blocked with a blocking solution of 10% normal goat serum (Thermo Fisher Scientific) and 1% BSA in PBS for 1 h. Tissue samples were incubated overnight at 4°C with the following primary antibodies: GPCR19 (Novus Biologicals), P2X7R (Clone 1F11, Biolegend), NLRP3 (Abcam), ASC (Santa Cruz Biotechnology), NeuN (Clone A60, Merck Millipore, Temecula, CA, USA), and GFAP (Thermo Fisher Scientific). Slices were subsequently incubated for 1 h at room temperature with Alexa 488-, 532-, or 546-conjugated IgG secondary antibodies, as appropriate, and then counterstained with DAPI for 10 min.

For apoptotic cell (Tunnel+) detection, frozen tissue sections from TDCA- or PBS-treated 5xFAD mice were fixed using 4% neutral buffer formalin in TBS for 20 min at RT. After washing, the TBS slides were incubated with 0.1% Triton X-100 for 3 min. The TUNEL assay was performed according to the manufacturer’s instructions (Roche, *In-Situ* Cell Death Detection TMR, red) and then counterstained with DAPI.

For Iba-1 (Wako, Osaka, Japan) staining, a frozen section was prepared from a separate group of TDCA- or PBS-treated 5xFAD or B6 mice. Perfused brains were cryopreserved in a phosphate-buffered 30% sucrose solution for 24 h. The brains were then frozen on dry ice, cut on a cryostat into sequential 3 μm sections, and stored at −80 °C until processed for analysis.

The Aβ core plaque was labeled by treating brain tissues with 1% thioflavin-S (Sigma–Aldrich) in PBS for 10 min at room temperature after being deparaffinized in xylene and rehydrated in a graded ethanol series (100%, 90%, 80%, and 70%). The tissue slides were then washed three times with 70% ethanol following DW three times. Fluorescence imaging was performed using a Confocal Microscope A1 (Nikon). NIS-Elements.AR. Ink (version 4.2, Nikon) was used to measure the MFI of the ROI.

### Global Protein Profiling

Anesthetized mice were perfused with cold PBS, and the whole brain was extracted, followed by snap-freezing using liquid nitrogen. The frontal cortex and hippocampus region of each mouse were collected and digested using urea- (8 M) based in-solution digestion. After protein quantification using the BCA assay (Micro BCA Protein Assay Kit, Thermo Fisher Scientific, Bremen, Germany), samples were pooled together group by group, and 300 µg of tissue extracts per group was separated *via* high pH reversed-phase fractionation using an Agilent 1260 HPLC infinity purification system (Agilent Technology, Santa Clara, CA, USA). The fractionated peptide samples were subsequently loaded onto traps (C18, 3 μm, 0.7 cm, Thermo Fisher Scientific) and EASY-Spray columns (C18, 2 µm, 100 Å, 50 cm, Thermo Fisher Scientific). Easy nano II Ultra Performance Liquid Chromatography and Q-Exactive Mass Spectrometry systems (Thermo Fisher Scientific) were used to separate the peptides. For Q-Exactive, a top 10 method was used. The Orbitrap mass analyzer was used to acquire full MS scans (m/z 300–1,600 range; resolution, 70,000). The Trans Proteomic Pipeline (Seattle Proteomic Center, Seattle, WA, USA) was used to convert the mass data files into mzXML files. Peptide masses were searched using a concatenated forward and reverse mouse international protein index (IPI) database (Decoy ipi.MOUSE.v3.80 database, 54285 entries) (63) with the SEQUEST-Sorcerer platform (Thermo Fisher Scientific, Sage-N Research, Milpitas, CA). Sorcerer (Sage-N Research, Milpitas, CA, USA) was used to estimate the FDR. Scaffold Q+ (Proteome Software, Portland, OR, USA) was used to compare spectral counts, validate MS/MS-based peptides, identify proteins (FDR < 1% in at least 2 peptides), and calculate Log2-fold changes (FC) and *P* values (Student’s *t*-test). Differentially expressed proteins (FC > 2, and *P* values < 0.05) were uploaded to the web-based Ingenuity Pathway Analysis (IPA^®^) software (Version 26127183; QIAGEN) for functional analysis and protein interaction networks.

### Statistical Analysis

The data are expressed as the mean ± SEM and were analyzed using a two-sided, unpaired Student’s *t*-test. The mean value between groups was compared using GraphPad Prism 5.0 software (GraphPad Software, La Jolla, CA, USA) unless otherwise indicated. *P* values < 0.05 were considered statistically significant.

## Data Availability Statement

The original contributions presented in the study are included in the article/**Supplementary Material**. Further inquiries can be directed to the corresponding author.

## Ethics Statement

The studies involving human participants were reviewed and approved by Korea Brain Bank Network (KBBN) operated through the National Brain Bank Project. The patients/participants provided their written informed consent to participate in this study. The animal study was reviewed and approved by Institutional animal care and use committees (IACUC) of Seoul National University (SNU-170517-25).

## Author Contributions

JI performed all experiments and assisted data analysis unless otherwise specified. J-AC set up all preliminary experiments. J-yK helped with the behavioral tests. K-SP helped with the Ca^++^ studies. Y-JK and CC helped in the water maze tests. E-JL, SN, KL, S-HK, S-HP, DL, BK, and K-HL provided the human samples for this project. S-YS conceived of and supervised the project, analyzed the data, and wrote the manuscript. All authors contributed to the article and approved the submitted version.

## Funding

This work was supported by a grant funded by the National Research Foundation, Ministry of Science, ICT and future planning (2012R1A5A2A44671346); a grant from the Korea Healthcare Technology R&D Project, Ministry of Health and Welfare (A062260); a grant from Gangwon Province, Republic of Korea; a grant from Chemon Co., Ltd. (0654-20170002); a grant from Shaperon Inc. (0654-20200002); and a grant for Shaperon from the Ministry of Health and Welfare (HI19C0429). The funders were not involved in the study design, collection, analysis, interpretation of data, the writing of this article or the decision to submit it for publication.

## Conflict of Interest

The patent “Pharmaceutical composition for prevention, treatment, or delay of Alzheimer’s disease or dementia containing G protein-coupled receptor 19 agent as active ingredient: CN106794189, EP3248603, ES2750839, IN201747003504, JP2017523982, KR1020150095951, US20180104261” was invented by Seong et al. and applied by Seoul National University R&DB Foundation. The exclusive license for the patent was transferred from SNU R&DB to Shaperon Inc. S-YS is a founder of Shaperon Inc. and its current CEO. Y-JK and CYC were employed by Shaperon Inc.

The remaining authors declare that the research was conducted in the absence of any commercial or financial relationships that could be construed as a potential conflict of interest.

## Publisher’s Note

All claims expressed in this article are solely those of the authors and do not necessarily represent those of their affiliated organizations, or those of the publisher, the editors and the reviewers. Any product that may be evaluated in this article, or claim that may be made by its manufacturer, is not guaranteed or endorsed by the publisher.
