# Supplementary material for: GPCR19 Regulates P2X7R-Mediated NLRP3 Inflammasomal Activation of Microglia by Amyloid β in a Mouse Model of Alzheimer’s Disease
Source: Front Immunol. 2022 Apr 6;13:766919. doi: 10.3389/fimmu.2022.766919 (PMC9019633; doi:10.3389/fimmu.2022.766919)
Supplement: Supplementary file 1 [file DataSheet_1.docx]

**Supplementary Figures**

**S Fig 1. a,** Representative gating strategies of flow cytometry to determine the purity of isolated primary microglia. DAPI^-^ CD11b^+^CD45^+^ cells were gated. **b,** Primary microglia isolated from B6, GPCR19^-/-^, and P2X7R^-/-^ mice were immune stained. Expression levels of GPCR19 or P2X7R were determined by measuring the MFI of ROIs in individual channels after staining with anti-GPCR19 antibody (green) and anti-P2X7R antibody (red). The nuclei were stained with DAPI (blue). Merged (GPCR19 (green) + P2X7R (red)) channels show co-localization area (yellow).

**S Fig 2.** Representative confocal images of individual channels from non-AD and AD patients were stained with anti-P2X7R (Red) and anti-GPCR19 (green) antibodies. The nuclei were stained with DAPI (blue). Merged (GPCR19 (green) + P2X7R (red)) channels show co-localization area (yellow).

**S Fig 3.** BV2 cells were treated with Aβ (2 µM), TDCA (400 ng/ml), and ATP (1 mM) for 1 h. Surface staining of cells showing GPCR19 (green) and P2X7R (red). The nuclei were stained with DAPI (blue). Merged (GPCR19 (green) + P2X7R (red)) channels show co-localization area (yellow).

**S Fig 4.** The frontal cortex of 5xFAD mice treated with TDCA (1 mg/kg, i.p., q.d.) for 10 weeks (n = 6/group) were analyzed using confocal microscopy after staining for GPCR19 (green) and P2X7R (red). The nuclei were stained with DAPI (blue). Merged (GPCR19 (green) + P2X7R (red)) channels show co-localization area (yellow).

**S Fig 5.** A gating strategy of flow cytometry to determine intracellular Ca^++^ in BV2 cells after staining with FURA2-AM. The amount of Ca^++^ in cells were analyzed by using Ca^++^ binding dye (FURA2-AM) for 300 sec. BzATP were added at 60 sec.

**S Fig 6.** Primary microglia cells were isolated and treated with Aβ (2 µM), TDCA (400 ng/ml) for 24 h, and ATP (1 mM) for the last 1 h. Individual channel of confocal microscopic slides shows NLRP3 (green) and ASC (red) expression levels. The nuclei were stained with DAPI (blue). Merged (NLRP3 (green) + ASC (red)) channels show co-localization area (yellow).

**S Fig 7.** Confocal microscopic slide of an individual channel for intracellular NLRP3 (green) and ASC (red) expression in the frontal cortex of 5xFAD mice treated with 1 mg/kg TDCA i.p., q.d. for 10 weeks. The nuclei were stained with DAPI (blue). Merged (NLRP3 (green) + ASC (red)) channels show yellow co-localization area and merged (NLRP3 (green) + ASC (red) + nuclei (blue)) channels show co-white colocalization area.

**S Fig 8.** 5xFAD mice were treated with TDCA (1 mg/kg i.p., q.d.) for 10 weeks and sacrificed. Representative confocal images of brains from 5XFAD mice show individual channel stained for DAPI (blue) and NeuN (red) (**a-d**). The frontal cortex (**a**), CA1 (**b**), DG (**c**) and CA3 (**d**) regions stained with NeuN are depicted. MFI ± SEM of specific ROIs (× 400) from **b** and **d** (left panel) were analyzed (n = 6 mice/group) in the right panel. **P* < 0.05 using Student’s unpaired *t*-test.

**S Fig 9.** Individual channel of confocal microscopy showing Iba-1^+^ (green) reactive microglia in frontal cortex (**a**) and in DG (**b**) of 5xFAD mice was treated with TDCA (1 mg/kg, i.p., q.d.) for 10 weeks. The nuclei were stained with DAPI (blue).

**S Fig 10.** Individual channel of confocal microscopy showing GFAP^+^ (red) reactive astrocytes in the cortex (**a**), DG (**b**), and CA3 (**c**) of 5xFAD mice treated with TDCA (1 mg/kg, i.p., q.d.) for 10 weeks. MFI ± SEM of specific ROI (× 400) from left panels (**a, b,** and **c)** were analyzed in the right panel (n = 9 mice/group). **P* < 0.05 using Student’s unpaired t-test.

**S Fig 11.** BV2 cells were treated with TDCA (400 ng/ml) for 12 h followed by incubation with fAβ (green) for 3 h. The phagocytosis of fluorescent Aβ (green) by BV2 cells was determined using confocal microscopy after staining with anti-LAMP-2 Ab (red).

**S Fig 12. a,** A schematic diagram showing protocols of behavior tests for 5xFAD and B6 (5xFAD^-^) mice after treatment with TDCA (1 mg/kg, i.p., q.d.) for 10 weeks. **b,** Changes in the body weight of mice were depicted. **c,** Times staying on the target platform during the probe test of MWM were compared between the groups of mice from three separate experiments (n = 20~22/group). **P* < 0.05 using Student’s unpaired t-test.

**S Table 1. List of human brain tissue sections used in this study.**

| **KBBN ID** | **Gender** | | **Age** | **Brain** | **Disease** |
| --- | --- | --- | --- | --- | --- |
| Brains of individuals irrelevant with Alzheimer's diseases (Non-AD brains in the text) | | | | | |
| 06006009 | Male | 50-59 | | Hippocampus | Normal (No neuritic plaque; Stage 0, B0**) |
| 06005691 | Male | 60-69 | | Hippocampus | Normal (No neuritic plaque; Stage 0, B0**) |
| 01000633 | Female | 60-69 | | Primary motor cortex | Interstitial lung diseases |
| Brains of patients with Alzheimer's diseases (AD brains in the text) | | | | | |
| 01002998 | Female | 80-89 | | Hippocampus | Stage V, B3^**^ |
| 03002487 | Male | 80-89 | | Hippocampus | Stage VI, B3 |
| 05007905 | Female | 50-59 | | Brain cortex | Alzheimer's diseases neuropathological change |
| 05007911 | Male | 80-89 | | Brain cortex | Alzheimer's diseases neuropathological change |
| 01002964 | Female | 80-89 | | Primary motor cortex | Stage V, B3 |

** NIA-AA score

**S Table 2. List of primers used in this study.**

| # | gene name | Primer |
| --- | --- | --- |
| 1 | mIL-1β-F | TTGTTGCTGTGGAGAAGCTGT |
| 2 | mIL-1β-R | AACGTCACACACCAGCAGGTT |
| 3 | mIL-12-F | GGAAGCACGGCAGCAGAATA |
| 4 | mIL-12-R | AACTTGAGGGAGAAGTAGGAATGG |
| 5 | mIL-10-F | GGTTGCCAAGCCTTATCGGA |
| 6 | mIL-10-R | ACCTGCTCCACTGCCTTGCT |
| 7 | mCD47-F | TTGCGAAGTGACAGAG TTATCC |
| 8 | mCD47-R | ACCTCCTTTCTCCTCCTCGTAA |
| 9 | mCD36-F | GAACCACTGCTTTCAAAAACTGG |
| 10 | mCD36-R | TGCTGTTCTTTGCCACGTCA |
| 11 | mSRB1-F | TTTGGAGTGGTAGT AAAAAGGGC |
| 12 | mSRB1-R | TGACATCAGGGACTCAGAGTAG |
| 13 | mIFN-γ-F | AACGCTACACACTGCATCTTGG |
| 14 | mIFN-γ-R | GCCGTGGCAGTAACAGCC |
| 15 | mTNF-α-F | AGCAAACCACCAAGTGGAGGA |
| 16 | mTNF-α-R | GCTGGCACCACTAGTTGGTTGT |
| 17 | mCCL11-F | TCCACAGCGCTTCTATTCCTG |
| 18 | mCCL11-R | GGAGCCTGGGTGAGCCA |
| 19 | mCCL17-F | CAGGGATGCCATCGTGTTTC |
| 20 | mCCL17-R | CACCAATCTGATGGCCTTCTT |
| 21 | mFPR2-F | ATTGTTGCTGTTTGCTATGGAC |
| 22 | mFPR2-R | CTGCTGTAAGGAC TCGTAAAGG |
| 23 | mSRA-F | ACATCACCAACGACCTCAGACT |
| 24 | mSRA-R | AGTTTGTCCAGTAAGC CCTCTG |
| 25 | mGAPDH-F | CAGTGGCAAAGTGGAGATTGTTG |
| 26 | mGAPDH-R | CTCGCTCCTGGAAGATGGTGAT |
| 27 | mIL-33-F | GGAGAAGGTGATGGTGAAC |
| 28 | mIL-33-R | CCACAACATCGTAAGCCAAG |
| 29 | mCCL5-F | AGATCTCTGCAGCTGCCCTCA |
| 30 | mCCL5-R | GGAGCACTTGCTGCTGGTGTAG |
| 31 | mNLRP-3-F | AGAAGAGACCACGGCAGAAG |
| 32 | mNLRP-3-R | CCTTGGACCAGGTTCAGTGT |
| 33 | mASC-F | GAGCAGCTGCAAACGACTAA |
| 34 | mASC-R | GCTGGTCCACAAAGTGTCCT |
| 35 | mPro-Caspase-1 F | GACTAAGTTGATTCCAAGCTC |
| 36 | mPro-Caspase-1 R | GATTCTGAGACCTACCTATG |
