# Supplementary figures and images for: GPCR19 Regulates P2X7R-Mediated NLRP3 Inflammasomal Activation of Microglia by Amyloid β in a Mouse Model of Alzheimer’s Disease
Source: Front Immunol. 2022 Apr 6;13:766919. doi: 10.3389/fimmu.2022.766919 (PMC9019633; doi:10.3389/fimmu.2022.766919)

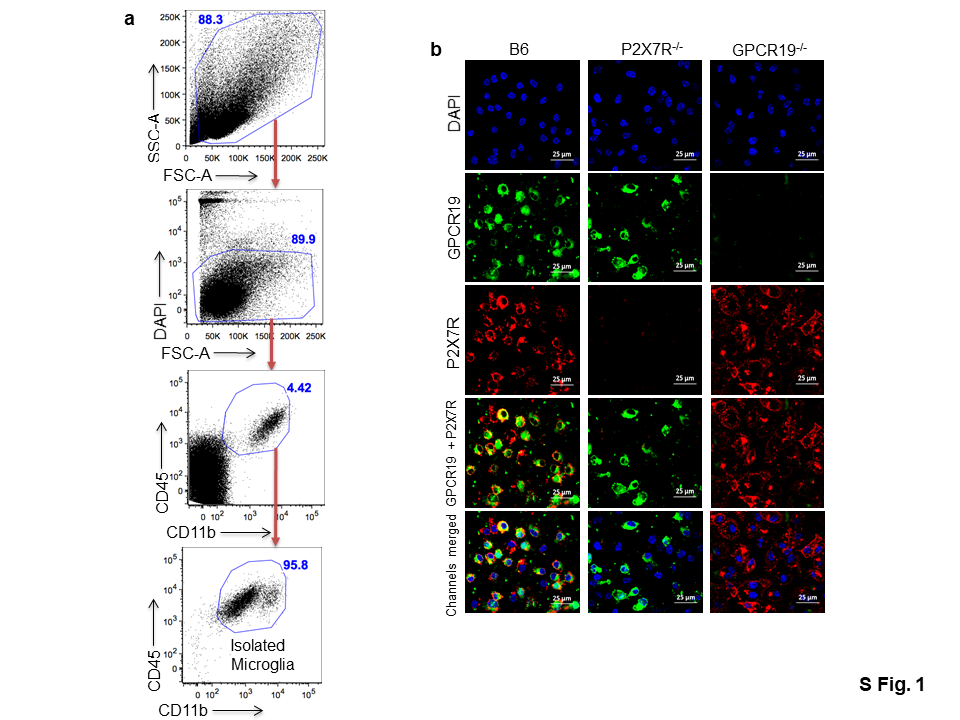

Supplement: Supplementary file 2 [file Image_1.tif]

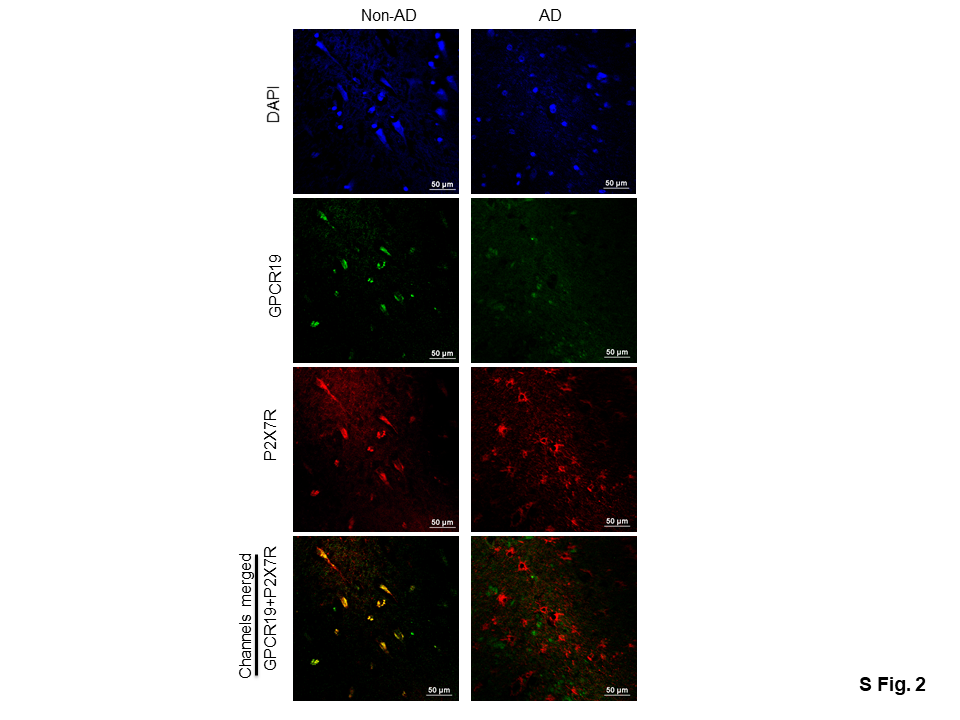

Supplement: Supplementary file 3 [file Image_2.tif]

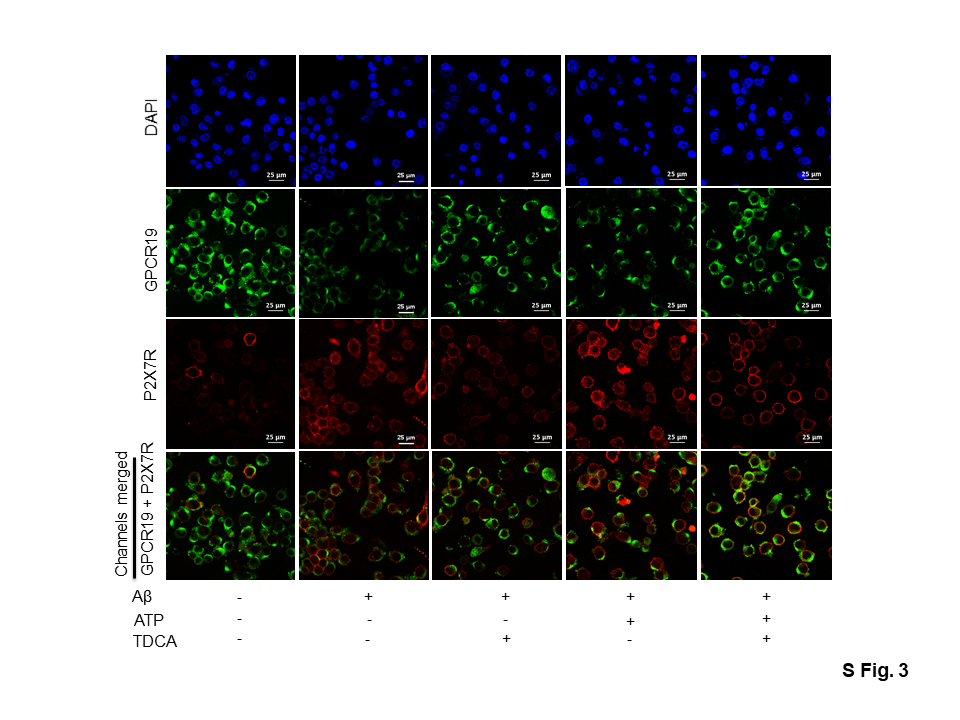

Supplement: Supplementary file 4 [file Image_3.tif]

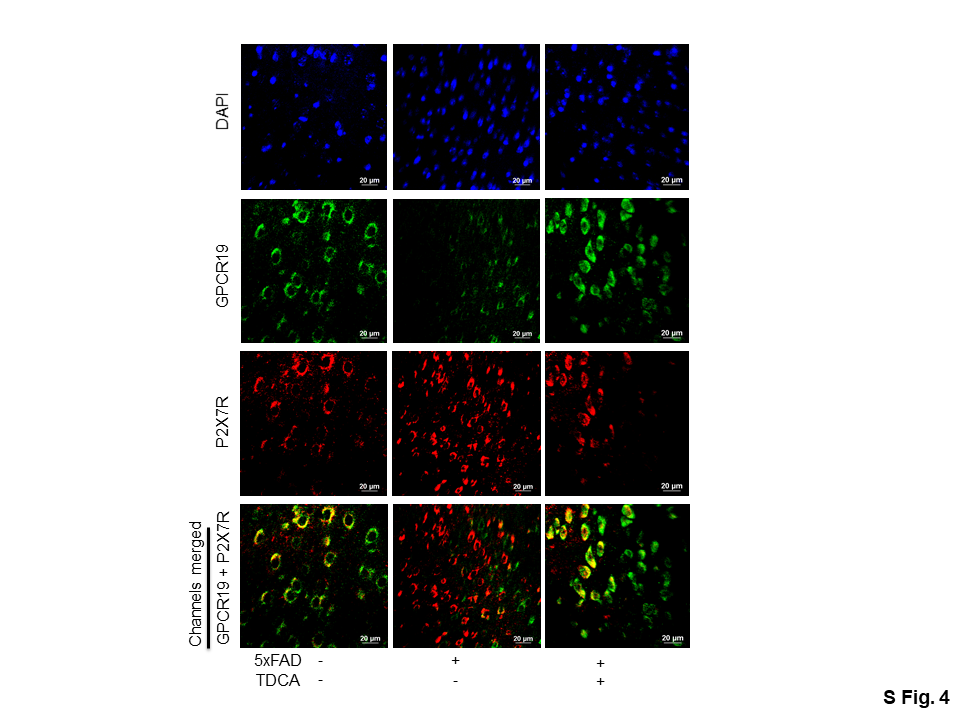

Supplement: Supplementary file 5 [file Image_4.tif]

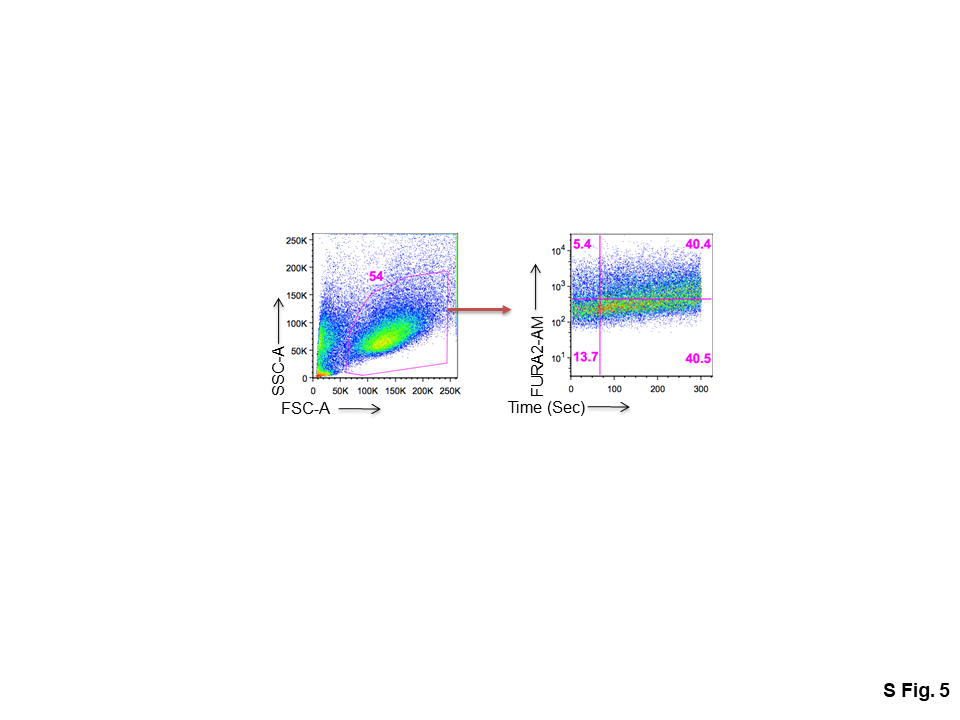

Supplement: Supplementary file 6 [file Image_5.tif]

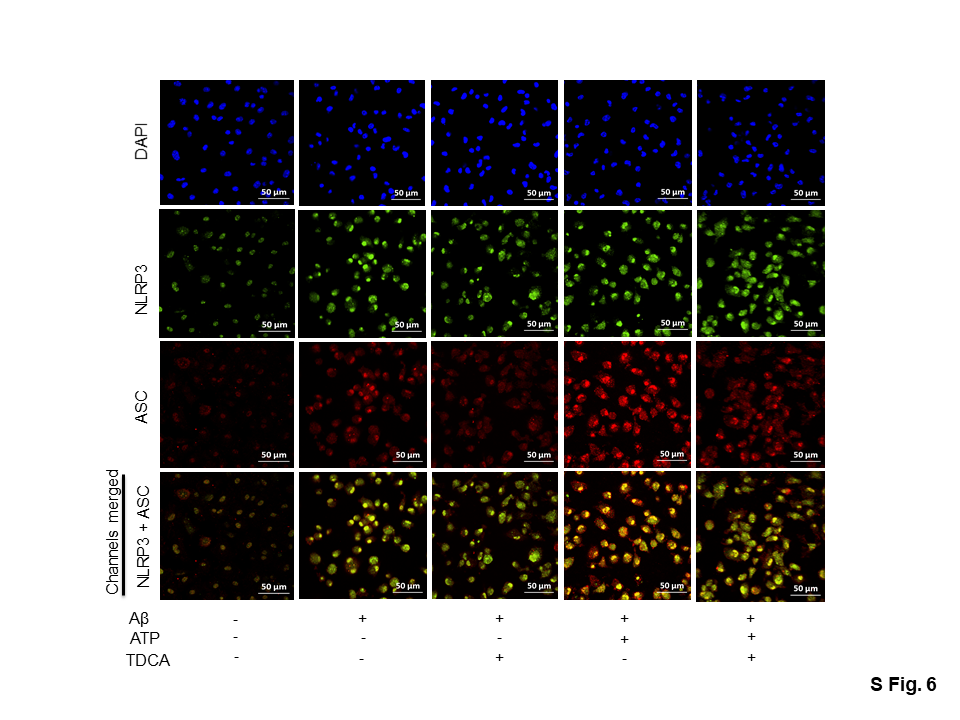

Supplement: Supplementary file 7 [file Image_6.tif]

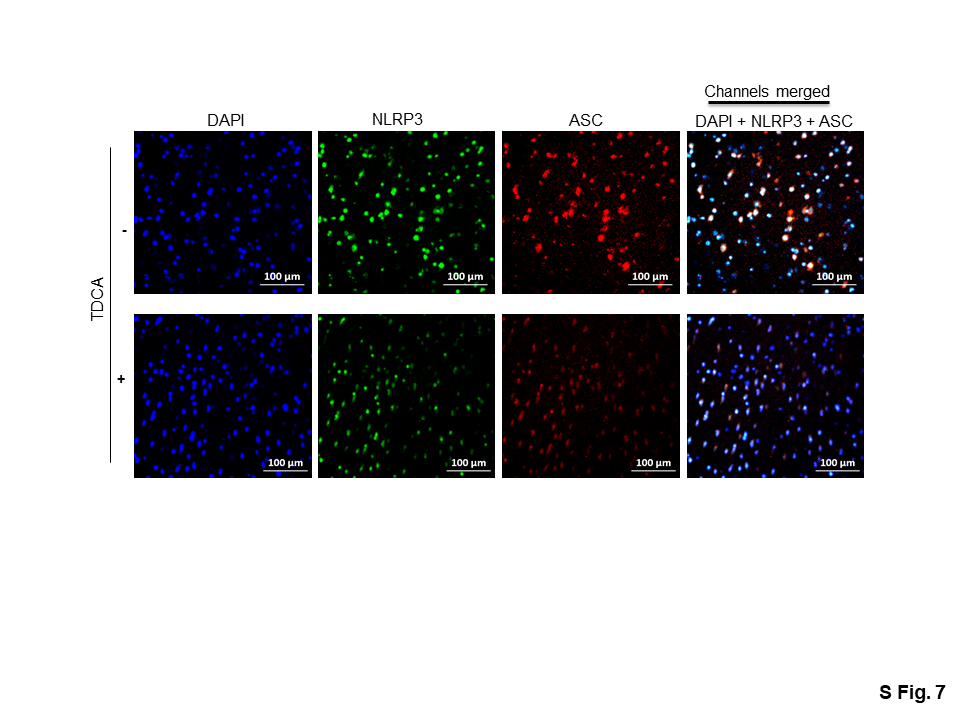

Supplement: Supplementary file 8 [file Image_7.tif]

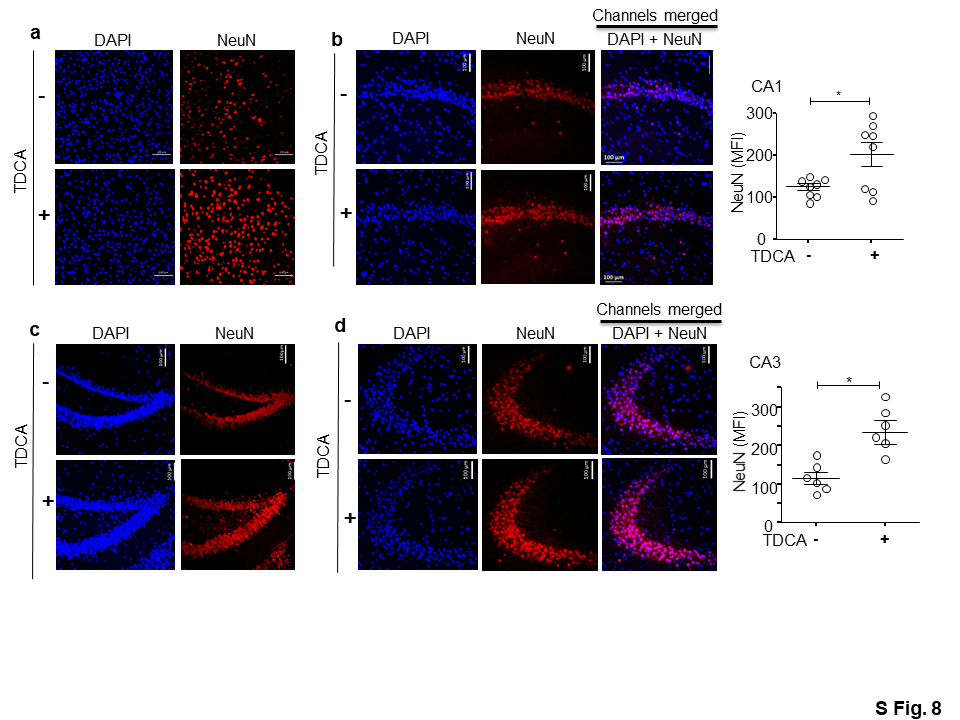

Supplement: Supplementary file 9 [file Image_8.tif]

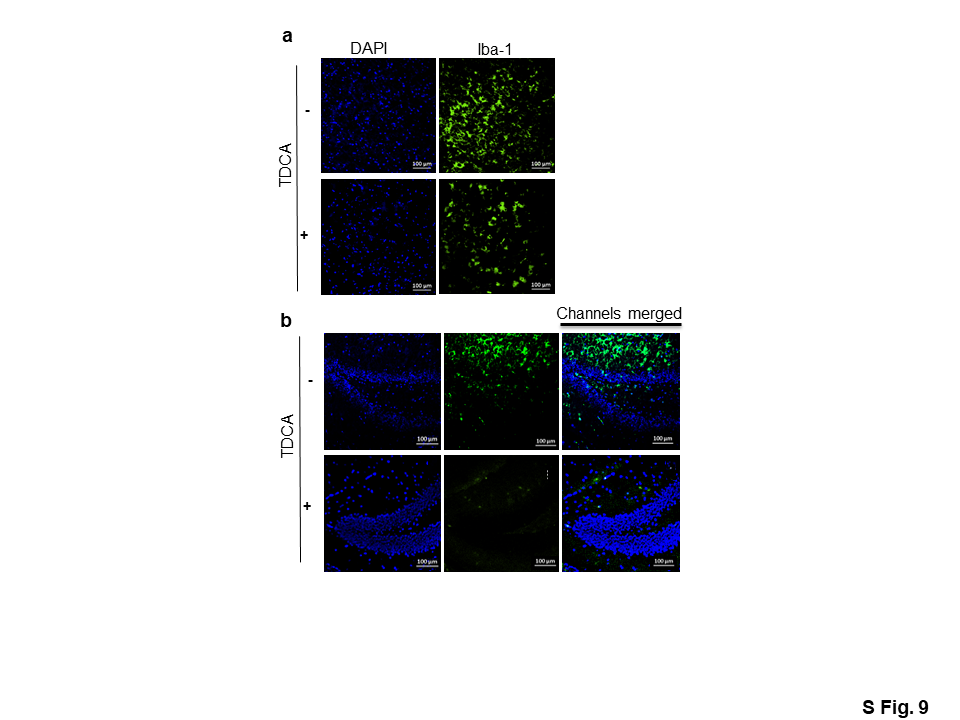

Supplement: Supplementary file 10 [file Image_9.tif]

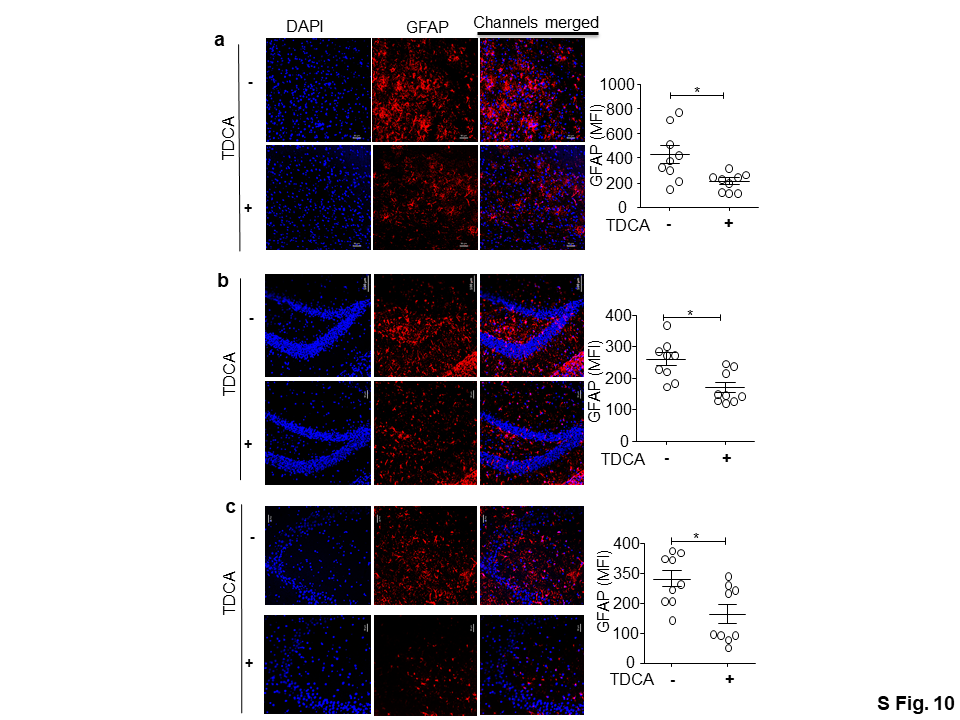

Supplement: Supplementary file 11 [file Image_10.tif]

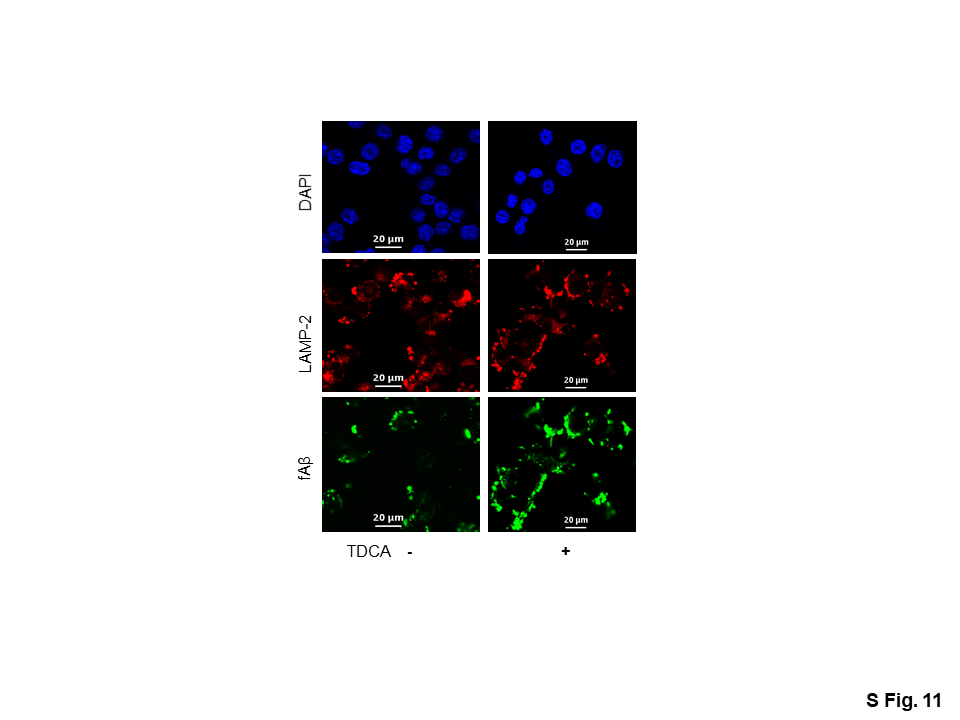

Supplement: Supplementary file 12 [file Image_11.tif]

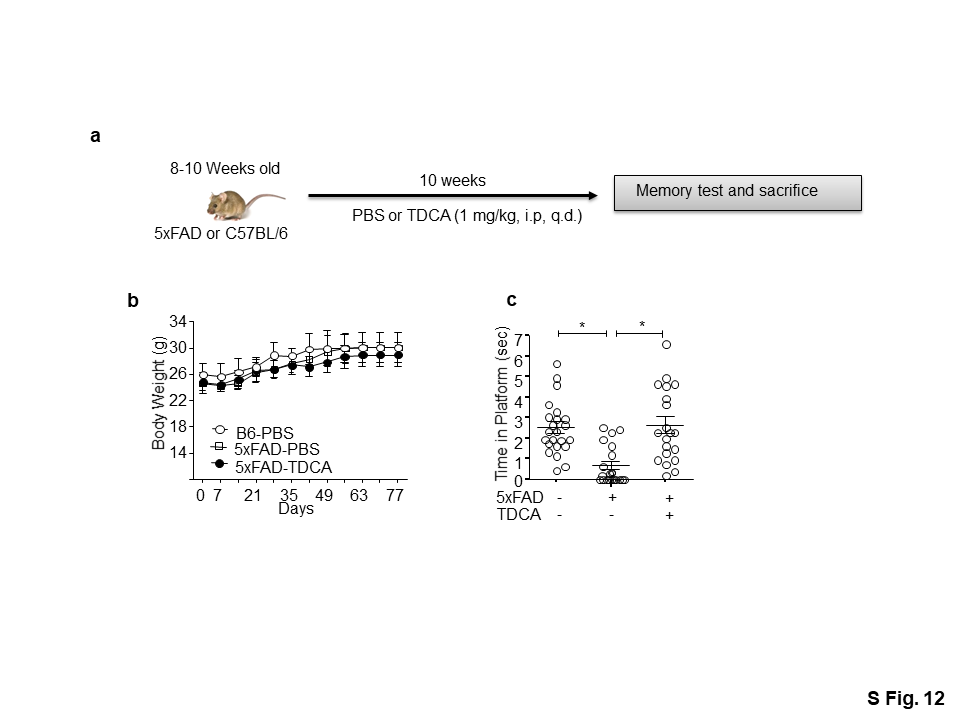

Supplement: Supplementary file 13 [file Image_12.tif]
